# Host specificity in vascular epiphytes: a review of methodology, empirical evidence and potential mechanisms

**DOI:** 10.1093/aobpla/plu092

**Published:** 2015-01-06

**Authors:** Katrin Wagner, Glenda Mendieta-Leiva, Gerhard Zotz

**Affiliations:** 1 Universität Oldenburg, Institut für Biologie und Umweltwissenschaften, AG Funktionelle Ökologie, Carl-von-Ossietzky-Straße 9-11, D-26111 Oldenburg, Germany; 2 Smithsonian Tropical Research Institute, Apartado Postal 0843-03092, Balboa, Ancón, Panamá, República de Panamá

**Keywords:** Biodiversity, host bias, host preference, host specificity, specialization, structurally dependent plants, vascular epiphytes

## Abstract

Information on the degree of host specificity is fundamental for an understanding of the ecology of structurally dependent plants such as vascular epiphytes. Starting with the seminal paper of A.F.W. Schimper on epiphyte ecology in the late 19th century over 200 publications have dealt with the issue of host specificity in vascular epiphytes. We review and critically discuss this extensive literature. The available evidence indicates that host ranges of vascular epiphytes are largely unrestricted while a certain host bias is ubiquitous. However, tree size and age and spatial autocorrelation of tree and epiphyte species have not been adequately considered in most statistical analyses. More refined null expectations and adequate replication are needed to allow more rigorous conclusions. Host specificity could be caused by a large number of tree traits (e.g. bark characteristics and architectural traits), which influence epiphyte performance. After reviewing the empirical evidence for their relevance, we conclude that future research should use a more comprehensive approach by determining the relative importance of various potential mechanisms acting locally and by testing several proposed hypotheses regarding the relative strength of host specificity in different habitats and among different groups of structurally dependent flora.

## Abstract (precis)

A considerable number of plants depend on structural support of other plants. To understand their diversity and ecology, it is essential to know how strongly potential host species differ in their suitability as hosts. This review focuses on vascular epiphytes, i.e. structurally dependent plants that do not parasitize their hosts. Despite a longstanding interest in the topic, our knowledge on the strength of their host specificity is still scanty. This is arguably due to conceptual confusion, but also because of the large complexity of the study system, which turns quantifying host specificity in the field into a challenge.

## Introduction

The relative breadth of tolerance or utilization of a species along particular niche axes is one of the most fundamental topics in biology (Futuyma and Moreno 1988). Niche axes of interest may be climatic variables, abiotic resources, but also potential biotic interaction partners, e.g. the set of potential host species for a guild of host-dependent species. The degree of host specificity has been studied for many different interaction types, including antagonistic (e.g. herbivory, parasitism), mutualistic (e.g. pollination, mycorrhizae) and commensalistic (e.g. epiphytism) ones.

Understanding the degree of host specificity of such host-dependent species is an important piece of the ‘diversity jigsaw puzzle’ (Kitching 2006). In principle, the diversity of a host-dependent guild may be a function of the diversity of the host guild as suggested for herbivorous insects in tropical rain forests (Novotny *et al*. 2006). Strong host specificity opens the possibility of sympatric speciation and may allow species coexistence by niche complementarity. Determining the degree of host specificity is also important in a conservation context because specialist species are generally more vulnerable to habitat alterations and climate change (Clavel *et al*. 2011) than generalist species, and host specialists, in particular, are threatened by coextinction with their hosts (Tremblay *et al*. 1998; Colwell *et al*. 2012).

Specificity is generally assumed to be the result of trade-offs between adaptations that (i) allow organisms to cope with diverse environmental conditions or (ii) allow the exploitation of different resource types. Specialization and generalization (i.e. the evolutionary processes of decreasing or increasing niche axis breadth) depend on the environmental heterogeneity experienced by a population (Futuyma and Moreno 1988; Poisot *et al*. 2011; Kassen 2002). Evolutionary pressure for generalization should be high in spatially or temporally fine grained, heterogeneous habitats—i.e. whenever members of the same population are likely to encounter heterogeneous resources or environmental conditions. In contrast, whenever the spatio-temporal grain is large relative to the dispersal ability or longevity of individuals, specialization should occur. Active habitat or resource selection (i.e. preference or avoidance) may reinforce specialization when preference and performance bias are matching (Ravigné *et al*. 2009). The expected degree of ‘host’ specificity should also depend on the type of species interaction. Specialization may be reinforced by coevolution in (i) antagonistic relationships (e.g. plant–herbivore systems) because of an ‘arms race’ (Ehrlich and Raven 1964) and (ii) in the mutualistic plant–pollinator system because plants may evolve mechanisms to favour specialist pollinators which are more effective than generalists (Blüthgen *et al*. 2007). Commensalistic relationships lack such driving forces for coevolution.

Many plants use other plants (mostly trees) as hosts that offer substrate area, ‘a place in the sun’ and, in the case of mistletoes, also water and carbohydrates. Since tree species differ in many traits (e.g. bark properties and foliage density), the growing conditions for these structurally dependent plants may strongly depend on the particular host species. Host specificity might arise if trade-offs prevent structurally dependent plant species to adapt equally well to all types of conditions found on different host species. Indications for a certain degree of host specificity have indeed been observed for all groups of structurally dependent plants: non-vascular (e.g. Barkman 1958; Palmer 1986) and vascular epiphytes (e.g. Callaway *et al*. 2002; Laube and Zotz 2006), hemiepiphytes (*sensu* Zotz 2013*a*) (e.g. Todzia 1986; Male and Roberts 2005), climbers (e.g. Putz 1984; Ladwig and Meiners 2010) and mistletoes (e.g. Hawksworth and Wiens 1996; Okubamichael *et al*. 2013).

Host tree specificity should be weaker for structurally dependent plants than for arboreal herbivores because in contrast to animals, plants cannot actively search for appropriate hosts and only have the options of establishing or perishing at the location where diaspores were carried by chance (Pennings and Callaway 2002). Based on theoretical considerations on coevolution under different interaction types, different groups of structurally dependent plants should show different degrees of host specificity. Mistletoes, climbers and hemiepiphytes tend to have an adverse effect on their host tree's fitness (e.g. Todzia 1986; Schnitzer and Bongers 2002; Aukema 2003). Thus, in these groups host specialization may be reinforced by an evolutionary arms race resulting in a high degree of host specificity. Indeed, a number of resistance mechanisms against mistletoe establishment have been described (Hoffmann *et al*. 1986; Yan 1993). For example, Medel (2000) presented evidence that mistletoe infection exerts a selection pressure for long spines, which reduce perching of mistletoe dispersers, in *Echinopsis chiloensis* (Cactaceae). Epiphytes, in contrast, being defined as rooting on their hosts without parasitizing them (Zotz 2013*b*), are generally assumed to have hardly any negative effect on their host trees. Consequently, comparatively weak host specificity is expected. Hypotheses can also be formulated regarding the degree of host specificity of structurally dependent plants in different forest types. In mixed forests in which no tree species is exceedingly abundant (as in tropical rainforests with typical hyperdispersed tree distribution patterns), structurally dependent plant species should have weak host specificity, because of strong selection to cope with these diverse conditions while in habitats with a few dominant tree species host specificity would be less penalized as diaspores of specialists have a greater chance of landing on or, in the case of climbers, near the host they are positively associated with (Barlow and Wiens 1977; Norton and Carpenter 1998; Nieder *et al*. 2001; Garrido-Pérez and Burnham 2010). Finally, in climates with a high diurnal or annual temperature variation and/or a pronounced dry season species have relatively broad climatic tolerances. This exaptation (*sensu* Gould and Vrba 1982) should be beneficial for coping with the range of microclimatic conditions found on different tree species.

**Figure 1.**
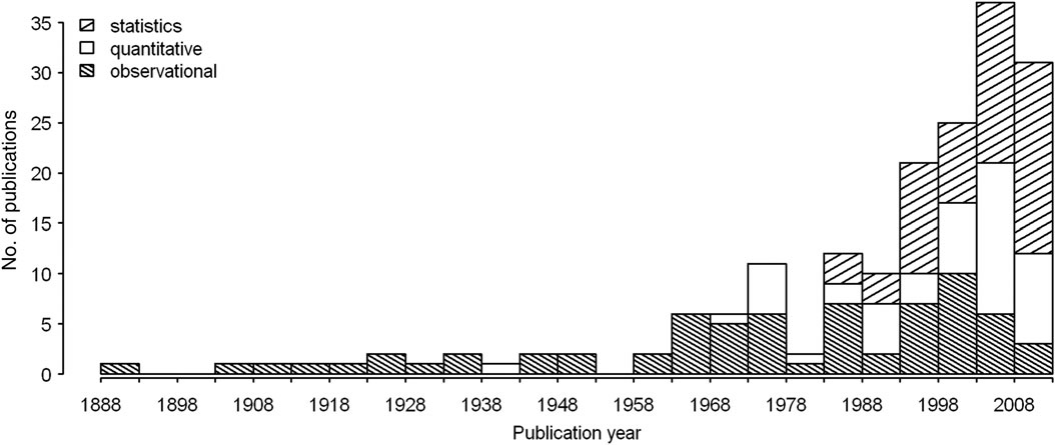
Histogram of publication dates (1888 through 2013 in 5-year intervals). Included were those publications, in which inference on host specificity of vascular epiphytes is based on own field observations. Excluded were studies that investigate mechanisms based on observations published in prior publications, articles only concerned with host specificity in the discussion section and secondary literature. Different shading lines indicate publication quality. Categories are: conclusions based on statistical tests (statistics), conclusions based on quantitative data (quantitative) and conclusions based on non-quantified observations (observational).

Vascular epiphytes represent ∼9 % of all vascular plant species (Zotz 2013*b*) and are a very important component of the plant assemblages of tropical wet forests (Gentry and Dodson 1987). However, notwithstanding their importance, our understanding of the mechanisms structuring epiphyte communities is still rather poor. Microclimate is a major determinant of the local distribution of vascular epiphytes as can be deduced from the vertical stratification of species documented in a large number of studies (e.g. Krömer *et al*. 2007; Zotz 2007). Host identity is another potential determinant, which has been invoked and/or investigated in >200 studies (Fig. 1, Appendix 1 **[see Supporting Information]**). However, while vertical gradients of microclimatic variables and epiphyte species distribution are relatively easy to document, the evidence for host specificity is much harder to obtain due to the complex vegetation structure and the multitude of candidate host traits.

With this review we pursue several goals. Definitions of terms are a prerequisite to ensure communication about concepts. In view of the ambiguous definitions of host specificity in many papers on vascular epiphytes, a terminology is proposed that precisely differentiates between different facets and assemblage-based scenarios of host specificity. Second, we provide a comprehensive overview on the empirical evidence for host specificity and on the potential proximate mechanisms that cause host specificity. Finally, investigating epiphyte host specificity has many pitfalls, and it is one of the major goals of this review to identify these problems, propose adequate approaches to solve them, promoting meaningful future field studies, which we discuss in detail in the final section of this paper.

## Terminology

For simplicity, ‘tree’ or ‘tree species’ are used when we refer to a potential epiphyte host individual or species although other growth forms (e.g. shrubs, lianas or columnar cacti) serve as epiphyte hosts as well. The terms ‘host’ and ‘host species’ imply that the focal epiphyte taxon has been observed on this tree individual or species. Note that, in unambiguous contexts, ‘host’ is often used as a short form of ‘host species’.

**Figure 2.**
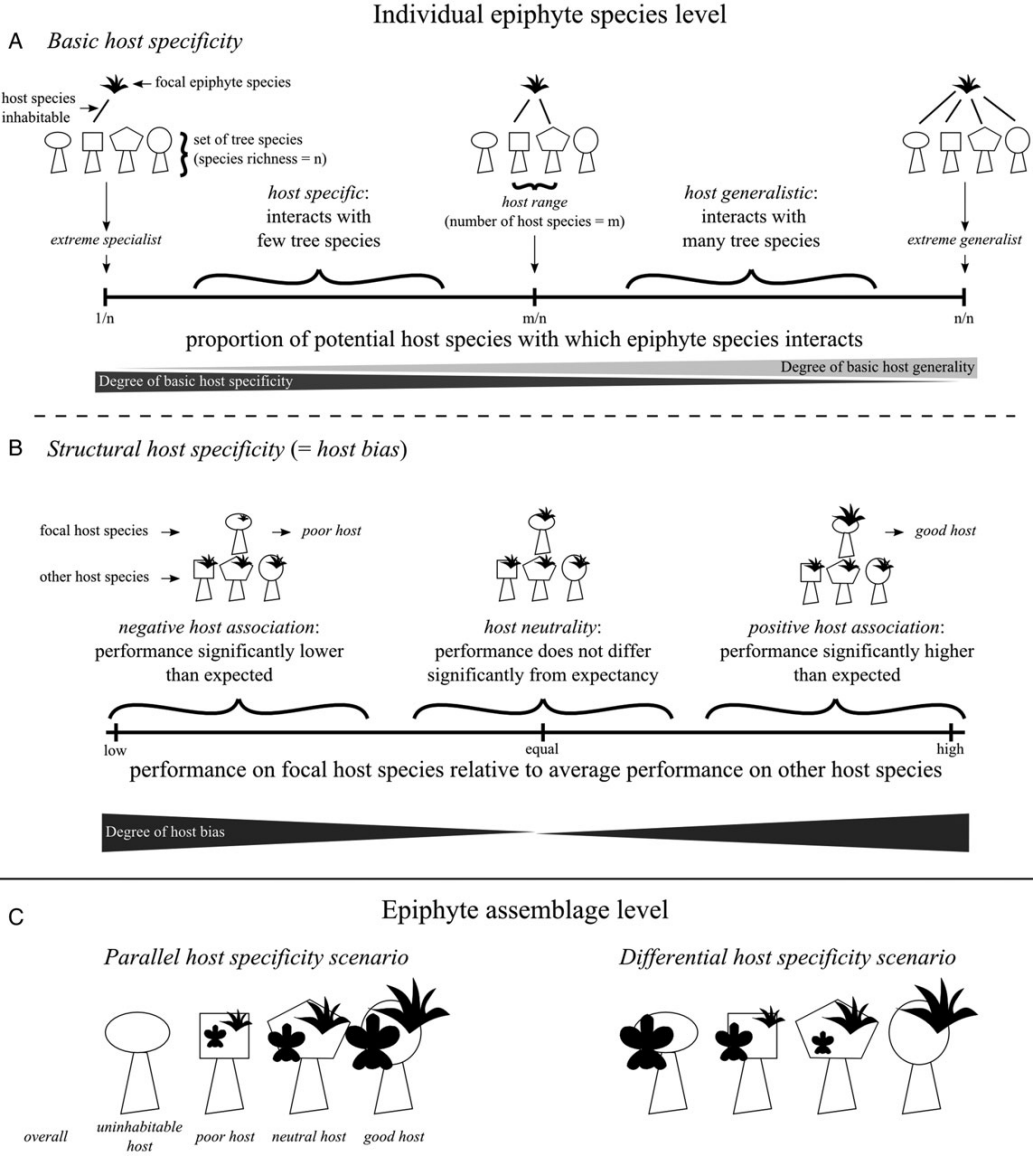
Terminology used throughout the text: (A) basic host specificity, (B) structural host specificity and (C) assemblage level scenarios. Different shapes represent different tree and epiphyte species, respectively. Epiphyte symbol size represents relative performance on a host species (measured as abundance, occupancy or fitness parameters of individual plants).

Ecological specificity can be separated into two components: the ‘range’ of occurrence along a niche axis and the ‘evenness’ of performance within this range (Devictor *et al*. 2010). In order to differentiate between these ‘facets of specificity’ we use the terms ‘basic’ and ‘structural host specificity’, which were introduced by Poulin *et al*. (2011). ‘Basic host specificity’ measures how many tree species are inhabitable by a focal epiphyte species (Fig. 2A). To allow comparison of figures from habitats with different numbers of tree species we suggest determining host specificity relative to the set of tree species. Depending on the scope of the study this may, for example, comprise all species occurring at the study site or throughout the distributional range of the epiphyte. As a proportion, basic specificity is a continuous trait ranging from ‘monospecificity’ (the epiphyte species can only inhabit a single tree species) over ‘intermediate basic specificity’ (it can inhabit a subset of tree species) to ‘complete basic generality’ (it can inhabit all tree species). The term ‘host range’ refers either to the raw number or to the set of host species. ‘Structural host specificity’ measures a ‘host bias’ (Gowland *et al*. 2013), i.e. differences in the performance (measured as occupancy, abundance or fitness parameters) of the focal epiphyte species on a given host species relative to other host species (Fig. 2B). The term ‘structural’ refers to the fact that population structure differs across host species (Poulin *et al*. 2011). When considering the association of a pair of epiphyte and tree species, the possibilities vary continuously from ‘uninhabitability’ over a ‘negative association’ and ‘host neutrality’ to a strong ‘positive association’. ‘Good’ and ‘poor host species’ are those on which epiphyte performance is disproportionally high or low. Note that, while all degrees of basic host specificity, with the exception of complete basic generality, imply structural host specificity, the reverse is not true: A species may be an extreme generalist concerning basic specificity (i.e. occur on all potential host species) and at the same time exhibit a host bias (with different performance on different host species).

Although basic and structural host specificity are closely interrelated and should both be quantified by metrics of specificity, it is instructive which of these facets of host specificity have been investigated in different contexts. For example, while studies on mistletoes are mostly concerned with basic host specificity, i.e. the specific set of utilized host trees (e.g. Downey 1998), most vascular epiphyte studies are concerned with structural host specificity, i.e. a performance bias among different host trees (e.g. Callaway *et al*. 2002). These different foci probably reflect the much stronger host specificity of mistletoes. Unfortunately, terms are inconsistently used in the literature on host specificity of vascular epiphytes. For example, in some cases host specificity was explicitly or implicitly defined as monospecificity, i.e. as the extreme scenario of an epiphyte species being exclusively associated with a single tree species (e.g. Mehltreter *et al*. 2005; Martínez-Meléndez *et al*. 2008). A term that has been repeatedly used to describe structural host specificity of structurally dependent plants is ‘host preference’ (e.g. Mehltreter *et al*. 2005; Laube and Zotz 2006; Martínez-Meléndez *et al*. 2008). However, since the terms ‘preference’ and ‘avoidance’ are reserved to the outcome of active host selection behaviour in the context of host–animal interactions (e.g. Futuyma and Moreno 1988; Desjardins *et al*. 2010; Takken and Verhulst 2013), we agree with Zhou and Hyde (2001) not to use these terms for sessile organisms.

When analysing host specificity patterns at the species assemblage level, it is necessary to distinguish two scenarios, although a mixture of both is expected in nature: (i) the ‘parallel host specificity scenario’, in which all epiphyte species show the same low or high performance on a given tree species, and (ii) the ‘differential host specificity scenario’, in which individual epiphyte species differ strongly in their performance on individual tree species (Fig. 2C). Differences in host quality in the parallel host specificity scenario are expressed with the terms ‘overall’ poor/good hosts.

## Investigating Epiphyte Host Specificity

### A bestiary of methodological approaches

Approaches to detect structural host specificity are diverse. For example, sampling designs differ greatly (plot- or transect-based sampling contrasts with the sampling of equal numbers of different tree species of standardized size). Similarly variable is the number of studied epiphyte species: it ranges from one or few focal species to the entire set of species at a study site or within a geographical region (Table 1). We classified studies according to the main types of response variables: (i) occupancy, (ii) abundance, (iii) species richness, (iv) species composition, (v) fitness parameters and (vi) network topology.

- Typically, only a fraction of trees in a forest is colonized by epiphytes and an even smaller fraction of trees is colonized by a particular epiphyte species. ‘Occupancy’ (i.e. presence/absence of epiphytes on a tree) is a widely used response variable in studies of host specificity (20 % of the 96 analyses in Table 1).
- Epiphyte ‘abundance’ per tree is the most commonly used response variable to test for host biases: 38 % of the analyses use a measure of abundance (Table 1). It can be measured as the number of individuals, biomass or cover per tree. The last measure, i.e. the proportion of the substrate area of a tree used by epiphytes, implicitly takes into account the usually positive relationship of epiphytic abundance and host size (see the following subsection on the pitfalls of the analyses). In some cases, the pooled abundance of all epiphytes was used as response variable (Table 1), which allows the identification of overall good or poor host species. However, the pooled epiphyte abundance allows no inference on host biases of single species under the differential host specificity scenario because biases of single epiphyte species might cancel each other out.
- ‘Species richness’, i.e. the number of epiphyte species per tree, has been used as a response variable in 15 % of the analyses (Table 1). However, this response variable underestimates host biases under the differential host bias scenario and is only indicative of host bias strength under the parallel host bias scenario.
- Epiphyte ‘species composition’ as response variable identifies differential host biases and was used as response variable in 11 % of the analyses (Table 1).
- ‘Fitness parameters’ (e.g. plant size, growth rate or physiological parameters) may be used to compare plant performance on different host species. This approach, which may be most useful for a mechanistic understanding of host specificity, was applied in very few studies (Callaway *et al*. 2001, 2002; Martin *et al*. 2007; Zhang *et al*. 2010; Gowland *et al*. 2011).
- Tests for host specificity based on network analysis are indirect. The response variables are measures of ‘network topology’ (e.g. number of links) with host specificity assumed to cause a deviation of observed from expected topology of a null-model. The links between species in such a bipartite network can be qualitative (presence/absence of interactions between species) or quantitative (frequency of interactions between species). All network analyses (11 % of analyses in Table 1) have been published very recently.

**Table 1.** Studies using statistical tests to detect structural host specificity. The table comprises all 55 publications that applied valid statistical tests for host biases. Methodological details of the literature search are described together with Appendix 1 **[see Supporting Information]**. The methods and outcomes of each test are specified separately if several tests were performed within a study, which leads to 97 entries.

References	Epiphyte taxa	Tree taxa	RV	Test	Size	Host bias	Per cent cases with bias	Comments
Ackerman *et al*. (1996)	1	16	ocs	8	No	Yes	100	Inference: confidence interval
Addo-Fordjour *et al*. (2009)	29	65	ric	3	No	Yes	n/a	
Aguirre *et al*. (2010)	21	2^1^	com	7	STA	Yes	n/a	*Sabal palmetto* vs. other trees; epiphytes and hemiepiphytes pooled
	21	2^1^	ric	6	STA	Yes	n/a	*Sabal palmetto* vs. other trees; epiphytes and hemiepiphytes pooled
Andrade and Nobel (1996)	1	22	ocs	1	Ind	Yes	100	Unoccupied tree species excluded from analysis
Andrade and Nobel (1997)	4	4	abs	1	(STA)	Yes	n/a	
Azemi *et al*. (1996)	6^1^	4	abs	3	No	Yes	17	Epiphytes pooled into taxonomic groups; response for some groups: cover
	n/a	4	cop	3	COV	Yes	n/a	
Benavides *et al*. (2011)	n/a	8	abs	6	OBS	Yes	5	Frequency of deviation from expectation (depending on plot): 0–20 %, mean = 5 %
	14	n/a	ocs	6	CAV, SUA	Yes	2	Frequency of deviation from expectation (depending on plot): 0–12 %, mean = 2 %
Bennett (1984)	2	6	abs	3	No	No	0	Methods not well described
Bennett (1987)	4	n/a	abs	3	Ind	Yes	25	Abundance/tree
	4	n/a	abs	3	STA	No	0	Abundance/stem
	4	3–5	abs	1	STE	Yes	25	
	4	3–5	abs	1	No	Yes	100	
	4	3–5	abs	1	DBH	Yes	100	
	4	3–5	abs	1	BAA	Yes	75	
Bernal *et al*. (2005)	1	18	abs	1	No	Yes	100	
	1	18	abs	1	CAV	Yes	100	
	1	18	abs	3	No	Yes	100	
Bittner and Trejos-Zelaya (1997)	52	4	com	7	No	Yes	n/a	Epiphytes and hemiepiphytes pooled
Blick and Burns (2009)	19	7	nec	6	No	No	n/a	
	19	7	nes	6	No	?	n/a	Weak evidence for nestedness
Boelter *et al*. (2011)	n/a	3	abp	3	No	Yes	n/a	Trees in monospec. plantations
	n/a	3	ric	3	No	(Yes)	n/a	Trees in monospec. plantations; no difference with richness estimators
Breier (2005)	22	12	com	7	No	Yes	n/a	
Burns and Dawson (2005)	6	7	ocs	2	DBH	Yes	33	
	20	7	ric	5	DBH	Yes	n/a	Significant interaction between dbh and host species
Burns (2007)	19	7	ned	4	OBS	No	n/a	Linear regression between observed and expected network degree
Burns and Zotz (2010)	77	8	ned	6	OBS	Yes	n/a	
	77	8	nes	6	No	?	n/a	No nestedness
	77	8	nec	6	No	Yes	n/a	
Callaway *et al*. (2001)	1	8	abs	3	(STA)	Yes	100	Essentially same results as in Callaway *et al*. (2002)
Callaway *et al*. (2002)	2	8	fit	3	n/a	Yes	100	Measurement of growth in transplants
Cardelús (2007)	53	2	cop	3	STA	Yes	n/a	
Castaño-Meneses *et al*. (2003)	1	2^1^	abs	3	(STA)	Yes	100	*Abies religiosa* vs. several *Quercus* species
Dejean *et al*. (1995)	5^1^	4	abs	1	No	Yes	60	Multiple comparisons problem; all *Tillandsia* pooled
Diaz Santos (2000)	71	47^1^	com	7	No	Yes	n/a	Phorophytes: genus level, two different analyses
Einzmann *et al*. (2015)	83	5	com	7	DBH	Yes	n/a	
	83	5	abp	3	(STA)	Yes	n/a	
	83	5	ric	3	(STA)	Yes	n/a	
	2	3	fit	3	n/a	Yes	50	
Fontoura (1995)	5^1^	14^1^	ocs	1	No	Yes	n/a	Bromeliads: genus level; trees: family level
	5^1^	14^1^	ocs	7	No	Yes	n/a	Bromeliads: genus level; trees: family level
García-Franco and Peters (1987)	6	5	abs	1	No	Yes	n/a	
Gowland *et al*. (2011)	3	5–10	ocs	1	No	Yes	100	Test especially designed for this study; number of woody species depends on site
	3	2^1^	fit	1,5	n/a	Yes	67	*Backhousia myrtifolia* vs. other trees; resp.: flower, leaf length, no. of inflor. and leaves
de Guaraldo *et al*. (2013)	1	11	ocs	1	No	Yes	100	
Hietz and Hietz-Seifert (1995*a*)	22–53	2–15	ric	5	BAA	(Yes)	n/a	Difference in only one out of six sites
	22–53	2–15	abp	5	BAA	(Yes)	n/a	Difference in only one out of six sites
Hietz and Hietz-Seifert (1995*b*)	39	n/a	ric	3	No	No	n/a	No. of tree taxa unclear
Hirata *et al*. (2009)	21	10	ric	5	DBH, HEI	Yes	n/a	
Köster *et al*. (2011)	381, 383	12, 11	ric	3	Ind	Yes	n/a	Species no.: 2 sites; trees: genus level (some)
Laube and Zotz (2006)	103	3	ocs	6	No	Yes	11	Depending on host species: 85–93 % of cases indistinguishable from chance
	103	3	abs	6	OBS	Yes	25	Depending on host species: 69–81 % of cases indistinguishable from chance
	43	3	com	7	No	Yes	n/a	Only 5 % of variance explained
Malizia (2003)	23	6	cos	6	COV	Yes	52	Epiphytes (18) and climbers (5) pooled, response: IV (occupancy + cover)
	23	6	com	7	COV	Yes	n/a	Epiphytes (18) and climbers (5) pooled
Martin *et al*. (2007)	1	3	fit	3	n/a	No	0	Chlorophyll concentration, CAM fluctuation, chlorophyll fluorescence
Martínez-Meléndez *et al*. (2008)	19	6	abs	1	BAA	Yes	100	Expected abundances based on ‘IV’ (host abundance + basal area)
Medeiros *et al*. (1993)	n/a	2^1^	abp	3	(STA)	Yes	n/a	Native vs. alien tree ferns
Mehltreter *et al*. (2005)	55	7	abp	3	No	No	n/a	
	55	7	ric	3	No	No	n/a	
	55	2	abp	3	STA	Yes	n/a	Tree ferns vs. angiosperms; dbh included by testing size classes separately
	55	2	ric	3	STA	Yes	n/a	Tree ferns vs. angiosperms; dbh included by testing size classes separately
	19	2	ocs	1	No	Yes	32	Tree ferns vs. angiosperms
Merwin *et al*. (2003)	n/a	3	abp	3	No	Yes	n/a	Trees in monospec. plantations; biomass per plot; host association depends on plot age
Moran *et al*. (2003)	31	2	ocs	1	STA	Yes	35	Tree ferns vs. angiosperms
	31	2	cos	3	STA	Yes	n/a	Tree ferns vs. angiosperms; tree size standardized by sampling comparable dbh
	31	2	ric	3	STA	Yes	n/a	Tree ferns vs. angiosperms; tree size standardized by sampling comparable dbh
Moran and Russell (2004)	1	2^1^	ocs	1	STA	Yes	100	*Welfia georgii* vs. dicot trees; tree size effect tested independently
	1	2^1^	cos	3	STA	Yes	100	*Welfia georgii* vs. dicot trees; tree size effect tested independently
Muñoz *et al*. (2003)	13	6	ocs	6	No	Yes	69	
	13	6	ric	6	No	(Yes)	n/a	Epiphytes and climbers pooled; richness deviating from expectation: 2 of 6 cases
Otero *et al.* (2007*a*)	1	22	ocs	1	No	Yes	100	Unoccupied tree species excluded from analysis; test performed for 2 sites
Piazzon *et al*. (2011)	5^1^	22	ned	4	No	Yes	n/a	Epiphytes and climbers pooled
	5^1^	22	nes	6	No	?	n/a	Nestedness higher than expected: in ca. 5–9 (depending on null-model) of 16 transects
Reyes-García *et al*. (2008)	6	n/a	abp	5	CAA, HEI	Yes	n/a	Explaining variable: leaf type (not host identity)
Roberts *et al*. (2005)	n/a	2	ocs	2	(STA)	Yes	n/a	Unclear how many epiphyte species tested
	97	2	ric	3	(STA)	Yes	n/a	Vascular and non-vascular epiphytes pooled
	97	2	com	7	(STA)	Yes	n/a	Vascular and non-vascular epiphytes pooled
Sáyago *et al*. (2013)	12	50	nes	6	No	?	n/a	Nestedness higher than expected
	12	50	nei	8	DBH	No	n/a	
Silva *et al*. (2010)	132	105	nes	6	No	?	n/a	
ter Steege and Cornelissen (1989)	56	2	abp	1	STA	Yes	n/a	
	56	2	ocs	1	STA	Yes	13	
Vergara-Torres *et al*. (2010)	6	10	abs	1	No	Yes	100	
	6	10	abs	1	BAA	Yes	100	
Wolf (1994)	521	31	com	7	COV	Yes	n/a	Vascular and non-vascular epiphytes pooled
Wyse and Burns (2011)	16	4	abp	1	SUA	Yes	n/a	
	16	4	cop	3	COV	No	n/a	
Zhang *et al*. (2010)	1	95	ocs	2	DBH	Yes	100	
	1	3^1^	fit	5	No	Yes	100	Hosts grouped in 3 association classes (bin. regression results); response: plant size
Zotz and Schultz (2008)	n/a	11	com	7	DBH	Yes	n/a	
Zotz *et al*. (2014)	15	6	ocp	1	No	No	n/a	
	15	2	ocp	5	DBH	No	n/a	

Abbreviations: n/a, not applicable or available.

Epiphyte taxa/Tree taxa gives the number of epiphyte or tree taxa included in the analysis.

RV notes the response variable used in the analysis. Categories: epiphyte abundance per tree–epiphyte species pooled (abp), epiphyte abundance per tree–single epiphyte taxa (abs), epiphyte species composition per tree (com), proportion of substrate area covered by epiphytes–epiphyte species pooled (cop), proportion of substrate area covered by epiphytes–single epiphyte taxa (cos), measure of fitness component or physiological parameter of epiphyte individuals (e.g. plant size, growth rate, chlorophyll fluorescence) rate (fit), network topology measure: checkerboard distribution, i.e. negative co-occurrence pattern in networks (nec), network topology measure: degree distribution (ned), network topology measure: interaction matrix (nei), network topology measure: nestedness, i.e. tendency for specialists to interact with perfect subsets of species interacting with generalists (nes), occupancy, i.e. presence/absence on tree–epiphyte species pooled (ocs), occupancy, i.e. presence/absence on tree–single epiphyte taxa(ocs), epiphyte richness, i.e. number of epiphyte species per tree (ric).

Test notes which kind of statistical test has been used. Categories: test for independence of variables in contingency tables: χ^2^ test, Fisher's exact test, *G*-test (1), response variable binary, explaining variable metric and/or nominal: Binary logistic regression, GLM (2), response variable metric, explaining variable nominal: ANOVA, Mann–Whitney *U*-test, Kruskal–Wallis test, *t-*test (3), response variable metric, explaining variable metric: Regression analysis (4), response variable metric, explaining variables: metric and nominal: ANCOVA, GLM (5), permutation tests (6), multivariate analyses: CA, CCA, Cluster Analysis, NMDS, PCA, SSHMS (7), other tests (8).

Size notes in which way problem of differential substrate area offered by different host species has been addressed. If appropriate gives proxy of tree size measured. Categories: basal area (BAA), canopy area (CAA), response data is per cent epiphyte cover, thus tree size already incorporated in response (COV), canopy volume (CAV), diameter at breast height (DBH), tree height (HEI), tree size not addressed in analysis of host specificity but independent test of correlation between tree size and response performed (ind), not necessary to account for size: response variable growth rate (n/a), tree size not addressed in analysis (no), permutation test, assumes that a tree can support the number of observed epiphyte individuals (OBS), size standardized by sampling design (STA), size roughly standardized by sampling design but still a lot of variation (STA), substrate area (SUA).

Host bias notes whether results indicate existence of non-random host epiphyte associations. Categories: analysis does not yield evidence for host bias (no), analysis yields evidence for host bias (yes), evidence for host bias mixed (yes), network metric, unclear how result should be interpreted in context of structural host specificity (?).

Per cent cases with bias gives percentage of tested epiphytes for which non-random distribution was found.

^1^Number does not refer to species level but to higher level taxonomic groups.

Numerous statistical methods are associated with this diversity of response variables (Table 1). Occupancy data are typically analysed with a test for independence of variables in a contingency table (like Pearson's χ^2^ or Fisher's exact test). Alternatively, some authors used binary logistic regression or permutation tests. Data on epiphyte abundance have been analysed with χ^2^ tests as well—but they also have been analysed, like richness and fitness data, with non-parametric ANOVA-style tests and general linear models with host identity as an explaining variable (these models may also incorporate size and other covariates). Species composition data call for multivariate statistics. Ordination methods like canonical correspondence analysis or cluster analysis have been employed. Resampling is typically used to test for deviations of observed network topology from the expected one—but occupancy, abundance and richness data have also been analysed with these methods.

### Pitfalls

Although the research question (‘Are epiphyte species distributed randomly among tree species?’) is simple, rigorously testing for structural host specificity is a difficult task because of the complex nature of species assemblages in the real world. Several critical factors should be taken into account when analysing data on epiphyte distribution to test for host specificity: most important are area and age of the substrate per tree species and clumped distribution patterns of epiphytes that are not caused by host specificity but by dispersal limitation.

Epiphyte occupancy and abundance per tree correlate axiomatically with two variables: tree age and substrate area (Zotz and Vollrath 2003). Longer exposure to epiphytic seed rain increases the probability of (repeated) colonization. Moreover, epiphyte accumulation rate should increase with tree age because young trees only accumulate epiphytes after diaspores successfully crossed the gap between their source host and the focal tree, while older trees will increasingly host reproductive epiphytes whose offspring is most likely to establish on the source tree (Zotz and Vollrath 2003). Similarly self-evident is the correlation with tree size: A larger tree will intercept more diaspores because it has a larger surface area exposed to the seed rain (Benzing 1990). However, the relationship between host size and age and epiphyte load is more complex. Larger trees within a forest stand will offer more diverse microclimatic conditions (Flores-Palacios and García-Franco 2006)—thus large trees may host additional species that do not find appropriate conditions on smaller trees. Certain host traits may change during tree ontogeny which, in turn, may lead to differential ‘life stage biases’. For example, Merwin *et al*. (2003) found that changes in host quality were related to ontogenetic changes in leaf size and deciduousness. Bark properties often change substantially with tree and branch age (Johansson 1974). The bark of young individuals and branches of trees with otherwise rough bark is often relatively smooth and thin (e.g. Everhart *et al*. 2009; Ranius *et al*. 2009). Extrinsic aging effects (bark weathering and the accumulation of non-vascular epiphytes and canopy soil) further change substrate quality (Catling and Lefkovitch 1989). Species-specific growth rates of trees, which are unknown for most field sites, add a complicating component to the study of structural host specificity because differential growth rates lead to different epiphyte loads at comparable tree sizes (Medeiros *et al*. 1993; Nieder *et al*. 2000; Hirata *et al*. 2009) or comparable tree age (Merwin *et al*. 2003).

Spatial autocorrelation is another issue. Many epiphyte species show clumped distributions (Nieder *et al*. 2000; Burns and Zotz 2010), which are primarily attributable to dispersal limitation (epiphyte offspring establishes most likely in proximity to the mother plant, i.e. on the same or nearby trees, Trapnell *et al*. 2004). Similarly, tree species may have a clumped distribution—even in highly diverse lowland rainforest habitats (Valencia *et al*. 2004).

When sampling design is plot-based or transect-based, tree species are sampled in unequal numbers. Additionally, size distributions of the sampled tree species differ, due to differential maximal sizes, longevity and growth rates but also stochasticity associated with low numbers of sampled trees per species in a typical field study. The limited number of sampled conspecific trees is particularly problematic in combination with clumped epiphyte and host distributions. Given data with such complexities, appropriate null expectations for the number of epiphytic individuals ‘per tree species’ should ideally be determined as functions of substrate area and age offered per tree species. In addition, spatial autocorrelation (clumped distribution patterns) should be considered. Note that the problem of spatial autocorrelation is alleviated when occupancy rather than abundance is used as response variable because in this case only the potentially clumped distribution of tree species (epiphyte diaspores land with a higher probability on neighbouring trees) may be a confounding factor.

Unfortunately, all these complexities and the associated problems in data analysis have been largely ignored in the past. For one, clumped distribution patterns and tree age have, so far, never been accounted for (Table 1). As the age of individual trees in a forest is typically unknown, tree size may be used as a compound proxy for size and age, although this approach is not ideal because growth rates generally differ among species. However, in 43 % of the published tests for structural host specificity (Table 1) only relative frequencies of tree species—but not tree size—were included (some of these studies performed separate analyses to test for a correlation between size and the respective response variable). Ignoring tree size may be acceptable when (i) tree species have comparable size distributions and (ii) large sample sizes preclude stochastic size differences. However, it becomes an issue whenever size distributions differ substantially between species (e.g. when the tree assemblage comprises understory shrubs, treelets and emergents). Researchers used different approaches to account for differences in tree size (Table 1). In some cases the size of the sampled trees or area was standardized. Alternatively, per cent substrate cover was used as response variable instead of numbers of individuals per tree. Finally, tree size was sometimes included as a covariate in statistical models or used to calculate expected occupancy or epiphyte load in contingency tables and permutation tests. Several proxies for tree size (e.g. dbh = diameter at breast height, tree height and canopy volume) were used. Arguably, substrate area is more directly linked to epiphyte load and occupancy than any of these proxies but has been only used in the exceptional case (Benavides *et al*. 2011; Wyse and Burns 2011).

## Empirical Evidence for Host Specificity

### Basic host specificity

While host range is of great interest in the case of mistletoes (e.g. Downey 1998), we are not aware of a single study that systematically quantified basic host specificity of vascular epiphytes. However, there are numerous observational accounts of epiphyte species that apparently grow exclusively on one host or several very closely related host species (‘extreme basic specificity’; Table 2).

**Table 2.** Studies reporting extreme basic host specificity.

References	Epiphyte species	Epiphyte family	Endemic	Host species/genus	Host family	Host life form	Habitat	Other trees?
Copeland (1916)	*Stenochlaena areolaris*	Blechnaceae	Yes	*Pandanus simplex*^1^	Pandanaceae	Tree	n/a	n/a
Cortez (2001)	*Trichomanes capillaceum*	Hymenophyllaceae	No	*Alsophila* spp.*, Cyathea* spp.*, Dicksonia* spp.	Cyatheaceae, Dicksoniaceae	Tree fern	n/a	Present
da Mota *et al*. (2009)	*Lepanthopsis vellozicola*	Orchidaceae	Yes	*Vellozia compacta*	Velloziaceae	Shrubby monocot	Outcrop	n/a
Hernández and Diaz (2000)	*Tetramicra malpighiarum*	Orchidaceae	Yes	*Malpighia* sp.	Malpighiaceae	Shrub	Forest	n/a
La Croix *et al*. (1991)	*Polystachya dendrobiiflora*	Orchidaceae	n/a	*Xerophyta* spp.	Velloziaceae	Shrubby monocot	Outcrop	n/a
Lecoufle (1964)	*Cymbidiella falcigera*^1^	Orchidaceae	Yes	*Raphia* sp.	Arecaceae	Palm	n/a	n/a
	*Cymbidiella pardalina*^1^	Orchidaceae	Yes	*Platycerium madagascariense*	Polypodiaceae	Epiph. herb	n/a	n/a
Li and Dao (2014)	*Coelogyne pianmaensis*	Orchidaceae	Yes	*Tsuga* spp.	Pinaceae	Tree	Forest	Present
Matias *et al*. (1996)	*Constantia cipoensis*	Orchidaceae	Yes	*Vellozia piresiana*, *Vellozia compacta*	Velloziaceae	Shrubby monocot	Outcrop	n/a
Mayo *et al*. (2000)	*Anthurium bromelicola* subsp. *bromelicola*	Araceae	Yes	*Hohenbergia* sp.	Bromeliaceae	Terr. herb	Outcrop	n/a
	*Anthurium bromelicola* subsp. *bahiense*	Araceae	Yes	*Aechmea* sp.	Bromeliaceae	Terr. herb	Coastal	n/a
Mesler (1975)	*Ophioglossum palmatum*	Ophioglossaceae	No	*Sabal palmetto*	Arecaceae	Palm	Forest	Present
Morris (1968)	*Polystachya johnstonii*	Orchidaceae	n/a	*Xerophyta splendens*	Velloziaceae	Shrubby monocot	Outcrop	n/a
Porembski (2003)	*Polystachya microbambusa*	Orchidaceae	No	*Afrotrilepis pilosa*	Cyperaceae	Shrubby monocot	Outcrop	n/a
	*Polystachya pseudodisa*	Orchidaceae	No	*Afrotrilepis pilosa*	Cyperaceae	Shrubby monocot	Outcrop	n/a
	*Polystachya odorata* var. *trilepidis*	Orchidaceae	Yes	*Afrotrilepis pilosa*	Cyperaceae	Shrubby monocot	Outcrop	n/a
	*Polystachya dolichophylla*	Orchidaceae	No	*Afrotrilepis pilosa*	Cyperaceae	Shrubby monocot	Outcrop	n/a
	*Pseudolaelia vellozicola*	Orchidaceae	n/a	n/a	Velloziaceae	Shrubby monocot	Outcrop	n/a
Schimper (1888)	*Trichomanes polypodioides*^1^	Hymenophyllaceae	No	n/a	n/a	Tree fern	Forest	n/a
	*Zygopetalum* sp.	Orchidaceae	n/a	n/a	n/a	Tree fern	n/a	n/a
Sehnem (1977)	*Zygopetalum maxillare*	Orchidaceae	n/a	n/a	n/a	Tree fern	n/a	n/a
	*Pecluma truncorum*	Polypodiaceae	n/a	n/a	n/a	Tree fern	n/a	n/a
Song *et al*. (2009)	*Thrixspermum odoratum*	Orchidaceae	Yes	*Quercus bawanglingensis*	Fagaceae	Tree	Forest	Present
Soto Arenas (1994)	*Laelia speciosa*	Orchidaceae	Yes	*Quercus deserticola*	Fagaceae	Tree	Forest	Absent
Tremblay *et al*. (1998)	*Lepanthes caritensis*	Orchidaceae	Yes	*Micropholis guianensis*	Sapotaceae	Tree	Forest	Present
Válka Alves *et al*. (2008)	*Vriesea oligantha*	Bromeliaceae	No	*Vellozia* spp.	Velloziaceae	Shrubby monocot	Outcrop	Present
	*Epidendrum saxatile*	Orchidaceae	No	*Vellozia* spp.	Velloziaceae	Shrubby monocot	Outcrop	Present
Van den Berg *et al*. (2006)	*Leptotes vellozicola*	Orchidaceae	Yes	*Vellozia* sp.	Velloziaceae	Shrubby monocot	Outcrop	n/a

Plant names follow *The Plant List* (2013, Version 1.1. Published on the Internet; http://www.theplantlist.org/).

n/a, not available (no information given in publication); Endemic, notes whether epiphyte species has a small distributional range; Other trees?, notes whether other potential hosts are present in the area to which publication refers.

^1^A different species name was used in the original publication.

In total, we found 28 reports of extreme basic specificity, most of which are species descriptions and/or purely observational (Appendix 1 **[see Supporting Information]**). Only two studies support their claim with quantitative data (Tremblay *et al*. 1998; Válka Alves *et al*. 2008). While a single field observation can prove the inhabitability of a tree species and thus ‘expand’ presumably limited host ranges, non-observations of epiphyte–tree interactions are only weak indicators for basic host specificity (Grenfell and Burns 2009). Thus, besides considering relative bark substrate areas of hosts and spatial autocorrelations, very large sample sizes are crucial to support claims of limited host ranges. Moreover, findings should be corroborated with experiments investigating exclusion mechanisms. To date, no claim of basic host specificity among vascular epiphytes satisfies these requirements.

The suspicion that many claims of extreme host specificity in epiphytes (Table 2) are sampling artefacts, or are only applicable to particular sites, is supported by a number of observations. Most species with allegedly extreme basic host specificity (71 %) are orchids. This is conceivably due to a possible host tree specificity of the symbiotic fungus (see the section on mechanisms), but rarity and localized populations of many orchids (Rogers and Walker 2002) offer alternative explanations. The study of few and small populations of an epiphyte species increases the likelihood that limitation to a single tree species is simply due to stochasticity in combination with host biases. A case in point is *Lepanthes caritensis*. This very rare orchid was found exclusively on the tree *Micropholis guyanensis* in the Carite State Forest, Puerto Rico, by Tremblay *et al*. (1998). However, later it was found on at least three other host species in another forest (Crain and Tremblay 2012). Similarly, while *Ophioglossum palmatum* may be monospecific in Florida (Mesler 1975), it colonizes other hosts in its distributional range (Ibisch *et al*. 1995; Cortez 2001). Finally, Válka Alves *et al*. (2008) report that the *Vellozia* ‘specialists’ at their study site grow on rocks elsewhere. However, there are cases in which independent observations corroborate a strong degree of basic host specificity. For example, the filmy fern *Trichomanes capillaceum* seems indeed to be restricted to tree ferns (Schimper 1888; Mickel and Beitel 1988; Cortez 2001; Mehltreter *et al*. 2005) as originally observed by Schimper (1888).

Porembski (2003) drew attention to rock outcrops in Africa and South America where shrubby or arborescent, mat forming monocots serve as hosts (several Velloziaceae and one Cyperaceae species). Almost half of all claims of extreme basic specificity refer to this type of vegetation (Table 2). Unfortunately, information on the diversity of potential hosts in this vegetation is largely missing (but see Válka Alves *et al*. 2008), opening the possibility that a lack of alternative hosts, rather than the inability to grow on other substrates, leads to the observed narrow host range. A blatant example where the reported extreme basic specificity should be attributable to the lack of alternative hosts is the case of *Laelia speciosa*, which occurs in monospecific forests (Soto Arenas 1994).

Interestingly, numerous cases of extreme basic host specificity feature hosts which are not the typical ‘tree’ growth form (Table 2). Notable examples are the mentioned shrubby monocots, tree ferns (five cases) and palms (two cases). There are also reports of an orchid that exclusively grows on an epiphytic fern (Lecoufle 1964) and on the association of an aroid with terrestrial bromeliads (Mayo *et al*. 2000)—although the aroid rather satisfies the definition of a miniature liana and its two subspecies associate with different bromeliad species.

### Structural host specificity

In almost all (89 %) of the 86 analyses that used statistical tests to detect structural host specificity (omitting network analyses because of their often unclear interpretation) authors rejected the null hypothesis of host neutrality (Table 1). Only nine analyses found no significant deviation from null expectations. While almost all analyses that focused on single epiphyte species found significant host biases (exception: Martin *et al*. 2007) this was not the case in analyses of entire epiphyte assemblages. Deviation from null expectations, i.e. a host bias, was only seen in a subset of the tested epiphyte species (47 ± 33 % SD; *n* = 11 studies that tested >4 epiphyte species and reported the number of host biases, Table 1). Presumably, studies that focus on few epiphyte species have higher statistical power (due to larger sample sizes per epiphyte species) to detect host specificity and/or the studied epiphyte species were selected based on prior observations that suggested host specificity.

In summary, we are not aware of any attempts to quantify potential basic host specificity in vascular epiphytes. Extreme basic host specificity seems to be an exception possibly restricted to especially demanding habitats, whereas host biases are ubiquitous among vascular epiphytes. However, we identified serious methodological issues (section on investigating epiphyte host specificity). Hence, future studies, which appropriately consider these complexities, might reveal that apparent host biases were, at least in some cases, methodological artefacts.

## Mechanisms

### Physical bark characteristics

Bark stability, texture and water-holding capacity are frequently hypothesized to be important for the suitability of trees as epiphyte hosts. The long-standing notion that trees with flaking or peeling bark are poor hosts is based on the assumption that such instable substrate hampers the establishment and survival of epiphytes (e.g. Schimper 1888; Went 1940). However, surprisingly few studies investigate this mechanism experimentally and no quantitative evidence of its importance exists. Schlesinger and Marks (1977) quantified ‘bark sloughability’ of branches with comparable diameter. They found that bark of host branches with higher bromeliad cover was more stable. However, the branches were also older and thus the effect of bark stability was possibly confounded by substrate age. Bark stability, just like other bark properties, may depend on tree ontogeny and plant part. For example, the bark of *Bursera fagaroides* peels with a higher rate on boles and large diameter branches as compared to small diameter branches and twigs (López-Villalobos *et al*. 2008). Surprisingly, the mortality of seedlings of two *Tillandsia* species was lower at locations with a higher peeling rate. Oliver (1930) stated that a number of New Zealand's conifers (among them the kauri, *Agathis australis*) are poor hosts due to their flaking bark. However, the crowns of mature kauris host many epiphytes (Ecroyd 1982), presumably because they offer a more stable substrate than trunks or branches of young trees.

Another common notion links bark roughness with the probability of epiphyte colonization, establishment and survival since rough bark offers a better foothold and thus prevents seeds and plants from being washed off. However, none of several correlative studies provide clear support for a mechanistic link (Migenis and Ackerman 1993; Otero *et al*. 2007*a*; Vergara-Torres *et al.* 2010). To our knowledge, the study of Callaway *et al*. (2002) is the only one to investigate how bark roughness influences epiphyte colonization in some detail. These authors quantified adherence of *Tillandsia usneoides* seeds and strands to bark of different host species and found that host biases, bark roughness and strand adherence were correlated. Nevertheless, correlations were weak because two species with relatively rugose bark (both pines) were poor hosts.

The capacity of bark to absorb (‘water-holding’) and temporarily store (‘water-retention’) rain water may increase host quality. Water-holding and retention capacity are functions of bark porosity (Johansson 1974) and thickness (Mehltreter *et al*. 2005). While Castro-Hernández *et al*. (1999) found no differences in final germination success on bark pieces and seedling mortality on trees with differential water-holding and retention capacity, several other studies do indicate that water-holding and/or retention capacity might play a role in host biases. For one, higher epiphyte loads of tree ferns could be explained by higher water-retention capacity and older age, as compared to angiosperms (Mehltreter *et al*. 2005). Similarly, when comparing two Tasmanian tree fern species, the species with higher water-holding capacity hosted significantly more epiphyte species (Roberts *et al*. 2005). Finally, size-corrected abundance of *T. usneoides* was positively correlated to bark water-holding and -retention capacities of 10 tree species (Callaway *et al*. 2002).

### Leaf and bark chemistry

Chemical properties of bark and leaves may also play a role in host specificity. Bark pH of tree species varies; different amounts of nutrients leach from leaves upon wetting and some tree species might exudate substances that act as allelochemicals.

In contrast to non-vascular epiphytes, for which bark pH has been strikingly often correlated to host biases (Barkman 1958; Löbel *et al*. 2006; Cáceres *et al*. 2007), bark pH has attracted only limited attention as a possible determinant of vascular epiphyte distribution and the few existing studies do not indicate any importance. Pessin (1925) detected no difference in pH of bark of hosts and non-hosts of the fern *Polypodium polypodioides*—although he noted that two pine species with low bark pH were completely devoid of the fern. The only recent pertinent study (Mehltreter *et al*. 2005) found no correlation of epiphyte abundance and bark acidity on the lower trunk either.

Epiphytes procure their nutrients either directly from atmospheric sources (dry and wet deposition), from throughfall and stemflow enriched with leaf and bark leachates or from tree and epiphyte litter (litter directly trapped by epiphyte structures and canopy soil). Although the proportions of tree and epiphyte litter in canopy soil are unknown, its chemical properties vary significantly between tree species (Cardelús *et al*. 2009). Assuming that leaf or bark leachates or tree litter play a significant role for epiphyte nutrition, host specificity may result from tree-specific differences (Benzing and Renfrow 1974). All studies that investigated the link of mineral nutrition and host biases focused on leaf leachates: Schlesinger and Marks (1977) determined the concentrations of minerals leached from foliage of three tree species, in throughfall and in bromeliad tissue in different forest types. Leachates of good hosts of *T. usneoides* were more enriched in minerals than those of poorer hosts, the throughfall concentrations of several minerals in different forest types correlated with bromeliad abundance, and the bromeliad tissue mineral concentrations reflected the forest types in a PCA. A quarter of a century later, Husk *et al*. (2004) were unable to replicate these results. This inconsistency may indicate that the host effect is modulated by changes in atmospheric nutrient inputs. A third study (Callaway *et al*. 2002) found only subtle effects of throughfall on *T. usneoides* and the fern *P. polypodioides*. Throughfall collected under good hosts increased the germination of *P. polypodioides* spores but did not increase epiphyte growth.

Trees might exude substances that are detrimental (allelopathic) to epiphytes. It was, e.g. hypothesized that tree species that exude latex are poor hosts for orchids (Piers 1968). Callaway *et al*. (2002) found that *T. usneoides* strands grew better when watered with rainwater as compared with throughfall collected below certain tree species. This contrasts with results from another study, in which strand elongation of mature *T. usneoides* was not influenced by leaf extracts (Schlesinger and Marks 1977). Allelopathic effects may be more important for earlier life stages. Indeed, several experimental studies found inhibitory effects of bark on germination and seedling performance: The inhibitory effect of different bark extracts on germination of *Tillandsia recurvata* seeds was negatively correlated with *in situ* epiphyte loads (Valencia-Díaz *et al*. 2010). In two older studies, the effect of powdered bark in the cultivation medium on germination and seedling development of the orchid *Encyclia tampensis* was tested (Frei and Dodson 1972; Frei 1973*a*). Expectations were based on differential orchid loads on these tree species in Mexico (Frei 1973*b*) and Florida. While differential inhibitory effects agreed quite well with field observations in Mexico, this was not the case for those from Florida. An important issue in the study of allelopathic effects is the concentration of potentially allelopathic substances. Arguably, the bark extracts used in experimental studies reach concentrations never occurring *in situ*.

### Architecture

Three important aspects of tree architecture are prevailing branch angles, diameter distribution of branches and leaf density. Dense foliage may buffer temperature and vapour pressure fluctuations and decrease the light intensity within canopies (Cardelús and Chazdon 2005). Additionally, it may decrease the amount of throughfall (Park and Cameron 2008)—especially during small rainfall events. Typically, dense foliage is assumed to have a negative effect on host quality. For example, Garth (1964) proposed that the strong interception of rain by the foliage of pines may partly explain why *T. usneoides* is underrepresented on them. However, the effect of dense foliage may be context-dependent. Gottsberger and Morawetz (1993) argued that in savannah habitats shade ‘favours’ epiphyte growth.

In many tropical forests deciduous and evergreen tree species co-occur, the proportion of deciduous species to evergreen species being correlated to the aridity of the respective site (Condit *et al*. 2000). During the leafless phase, usually coinciding with the dry season in the tropics, epiphytes experience more extreme microclimates on deciduous trees (Einzmann *et al*. 2015). The effect of deciduousness may depend on the particular epiphyte species. For xerophytic taxa like cacti (Andrade and Nobel 1997; Cardelús 2007) or some bromeliads (Birge 1911; Bennett 1987; Cardelús 2007) deciduousness may increase host suitability while it may decrease host suitability for more mesic species. Due to this filtering effect epiphyte richness should be lower on deciduous trees in tropical habitats (Einzmann *et al*. 2015). However, Hirata *et al*. (2009) found higher epiphyte richness on deciduous than on evergreen trees in a warm-temperate forest.

The prevailing branch inclination can influence the suitability of tree species via various mechanisms: (i) The danger of detachment and falling should be reduced if seeds or plants attach to horizontal as opposed to vertical substrate. (ii) Horizontal branches accumulate more canopy soil than branches with a steeper inclination (Nadkarni and Matelson 1991). An extreme example on how host architecture promotes the amount of accumulated canopy soil is the case of palms with persisting leaf bases. López Acosta (2007) found, on average, >9 kg (dry weight) of accumulated organic substrate per *Sabal mexicana* individual, a palm species that supported significantly more epiphytes and hemiepiphytes than non-palm trees of similar size (Aguirre *et al*. 2010). (iii) Branching angles may influence the ratio of stemflow to total precipitation (Park and Cameron 2008). Válka Alves *et al*. (2008) observed that water poured on trees from above ran down the trunk of two *Vellozia* species but ‘tended to fall vertically’ in other species being possibly responsible for the high epiphyte load of *Vellozia* species. Similarly, leaf traits (upward position and folding at night) have also been proposed to increase stemflow and dew formation and thus improve host quality (Wee 1978; Gottsberger and Morawetz 1993).

Conceivably, the relative distribution of branch diameters per tree species is involved in host specificity. Small diameter branches are more susceptible to mechanical damage and thus have higher turnover rates than large diameter branches (Watt *et al*. 2005). Therefore, tree taxa (like pines) lacking large diameter branches due to their branching pattern may be overall poor hosts (Garth 1964). Similarly, greater wood density could increase branch longevity and thus epiphyte load (Sáyago *et al*. 2013). Some epiphytes seem to depend on certain substrate diameters (Zimmerman and Olmsted 1992). For example, so-called twig epiphytes are found disproportionately on very small diameter branches (Catling *et al*. 1986). The underlying mechanism is unclear.

### Tree microhabitat

Sometimes the habitat of the host (i.e. its climatic niche) is viewed as a mechanism that causes host specificity. Arguably, it seems rather farfetched to conceive e.g. ‘boreal’ forest trees as poor hosts for ‘tropical’ orchids. However, it is a sensible perspective if tree species grow in distinctive locations within the same forest (e.g. Valencia *et al*. 2004; Kraft *et al*. 2008), and thus offer epiphytes specific microclimatic conditions. Annaselvam and Parthasarathy (2001) hypothesized that in their study plot *Chionanthus mala-elengi* was an overall good host ‘because of its occurrence in riverine areas’. Similarly, Mucunguzi (2008) observed that the ‘most preferred tree species occupied lower slopes and valleys near swamps’ in Kibale National Park, Uganda.

### Tree longevity and size at maturity

In the section on the investigation of host specificity, we pointed out that epiphyte occupancy and load generally increase with tree age and size and that ignoring these covariates may result in wrong conclusions on host specificity. However, tree species differ substantially in their longevity (e.g. live expectancy of trees in Central Amazonia ranges between 10 and 1000 years, Laurance *et al*. 2004) and their size at maturity (e.g. large size differences of mature understory shrubs and canopy trees) and these tree traits potentially influence host quality (e.g. Catling and Lefkovitch 1989; Wolf 1994; Annaselvam and Parthasarathy 2001; Bernal *et al*. 2005; Válka Alves *et al*. 2008).

Tree longevity and size at maturity may cause host biases via several mechanisms. First, there are the inherent age/size effects, i.e. accumulation probability increases with larger substrate area and longer substrate exposition. In this case host biases will only be seen if the ‘currencies’ in which epiphyte performance is measured are either occupancy or epiphyte abundance while individual plant fitness parameters do not depend on these effects. Other age and size effects should be observed with all performance currencies. Since exposed microsites may be limited to canopy tree species in forest stands, epiphytes specialized to such conditions have a low performance on understory tree species (Malizia 2003), albeit the same should be true for epiphytes on young individuals of canopy tree species (Zotz and Vollrath 2003). Moreover, canopy soil and other epiphytes may accumulate with time and may change the substrate quality on long-lived tree individuals.

### Epiphyte traits

Tree traits define host quality. However, ‘differential’ host specificity requires trait differences between epiphyte species. The candidate traits are numerous. One important set of traits are those that lead to microclimatic specificity. Vertical stratification of epiphyte assemblages is well documented and generally attributed to microclimatic gradients within vegetation (e.g. Zotz 2007). Epiphyte traits related to microclimatic specificity may cause differential host specificity if host species differ consistently in the microclimatic conditions they offer. Xerophytic epiphyte species are underrepresented on understory tree species while mesic species may be underrepresented on deciduous species (Einzmann *et al*. 2015). Other traits may allow particular epiphyte species to cope with substrate instability. For instance, the ability to entangle whole stems or branches with roots or stolons has been associated with successful establishment on trees with flaking bark (Schimper 1888; Benzing 1978). Fast life cycles and small size at maturity may be prerequisites to grow on host species with many small diameter branches while epiphytes that attain a large size may be restricted to host species that offer strong branches or large branch forks for mechanical support (ter Steege and Cornelissen 1989; Hemp 2001). Finally, diaspore characteristics and dispersal mode may also influence host specificity (Dejean and Olmsted 1997), e.g. via the ability to adhere to bark surfaces or via directed animal dispersal.

The demands of epiphytes for particular growing conditions may change during ontogeny, which may affect their host specificity. If, for example, seedling establishment is averted on a host species, it will be a poor host regardless of whether trees offer suitable growing conditions to later life stages (Went 1940; Dressler 1981). Zhang *et al*. (2010) found a positive correlation of plant size and height above the ground in *Asplenium nidus*, although this fern is most common in the understory. They concluded that microsites in the understory are more favourable for establishment, whereas the canopy offers better growth conditions for established individuals. Finally, host specificity may occur at different organizational levels. In this article we are mainly concerned with the species and community level but populations and even genotypes within populations may differ enough in their relevant traits as to show differential host specificity.

### Cascading effects

Host traits may not only have direct effects on vascular epiphyte performance but also act indirectly via their effect on other organisms (cascading effects, Thomsen *et al*. 2010). Associations of non-vascular epiphytes with host species are well established (e.g. Barkman 1958 and references therein, Gauslaa 1985; Palmer 1986; Loppi and Frati 2004). The colonization of trees by non-vascular epiphytes (bryophytes and lichens) may influence substrate suitability for vascular epiphytes and, thus, cause host biases (Pollard 1973; Sanford 1974; Zotz 2002). On the one hand, non-vascular epiphytes may facilitate the establishment of vascular epiphytes by storing water (Tremblay *et al*. 1998; Watthana and Pedersen 2008), enabling the accumulation of canopy soil (Watthana and Pedersen 2008), helping diaspores to adhere to the substrate (Callaway *et al*. 2001) and via the leaching of nutrients (Tremblay *et al*. 1998). On the other hand, they may decrease substrate suitability by competing for space (Zotz and Andrade 2002) or via allelopathic effects (Callaway *et al*. 2001). Repeatedly, authors have observed correlations between species abundances of non-vascular and vascular epiphytes (Van Oye 1924; Tremblay *et al*. 1998; Callaway *et al*. 2001; Zotz and Vollrath 2003; Watthana and Pedersen 2008; Gowland *et al*. 2011). However, experiments like the ones conducted by Callaway *et al*. (2001) are needed to assess whether these correlations are caused by cascading effects or are simply due to parallel effects of host traits on both groups.

It is also conceivable that host specificity is mediated by mycorrhizal fungi (Went 1940). Such a cascading effect suggests itself in the case of orchids (Clements 1987; Hietz and Hietz-Seifert 1995*a*; Gowland *et al.* 2011, 2013) which depend on the occurrence of symbiotic fungi for successful germination and seedling development (Arditti 1992). Some orchid species show specificity for certain fungal clades (Otero *et al*. 2002, 2004, 2007*b*). Moreover, the distribution of orchid mycorrhizal fungi is conceived to be independent of the distribution of orchids and may depend on tree species (see Gowland *et al*. 2013 and references therein). However, Gowland *et al*. (2013) could not confirm that the distribution of clades of mycorrhizal fungi (isolated from orchid roots) on host trees fully explains the observed host tree biases of three epiphytic orchid species.

Animals may influence epiphytic distribution patterns (Perry 1978). Zoochorous seeds may land disproportionately often on host species that are attractive to their dispersers due to the fruits or suitable perching sites they offer. This mechanism has already been suggested for hemiepiphytic figs (Guy 1977) and mistletoes (Roxburgh and Nicolson 2005). A special case is the mutualistic association of epiphytes with arboreal ants. For example, Kiew and Anthonysamy (1987) noted that the high epiphyte occupancy of *Gluta aptera* coincided with a high occupancy by ants. Similarly, Davidson (1988) attributed the biased occurrence of ant garden epiphytes on certain tree species to their extrafloral nectaries and location in disturbed areas (presumably correlated with an increased resource supply rate by phloem-feeding homoptera). Dependence on host tree traits is reduced if epiphytes establish in ant gardens (Benzing 1990). For example, the mediation of ants allows epiphytes to grow on smooth, short-lived giant bamboo culms (Kaufmann *et al*. 2001). Termites may play a similar role by allowing epiphyte establishment on their carton-runways (Flores-Palacios and Ortiz-Pulido 2005), although epiphytes inhabited their runways less frequently than the nests of arboreal ants (Blüthgen *et al*. 2001). The presence of arboreal ants might also reduce ‘host quality’: Ants, when living in association with myrmecophytic tree species (e.g. *Cecropia* spp.), might prune their hosts from establishing epiphyte seedlings (Janzen 1969). Janzen’s suggestion has been tested for vines (Janzen 1974; Fiala *et al*. 1989) but not yet for epiphytes.

### Climate, potential host pool and the spatial scale

Host biases are frequently inconsistent over large areas (Schimper 1888; Sanford 1974 and references therein, Sehgal and Mehra 1984). This phenomenon has been dubbed ‘regional phorophyte specificity’ (Ibisch *et al*. 1995) and seems to be similarly common among non-vascular epiphytes (Barkman 1958; Patiño and Gonzalez-Mancebo 2011). There are a number of possible reasons. The effects of the tree traits that potentially influence epiphyte performance are likely to be modulated by climate. For example, while a low bark water-retention capacity of a given tree species may render it a poor host in a xeric habitat, the same tree species may be a good host in a mesic habitat. Host traits might also differ geographically. Schimper (1888) observed that densely foliated Mango trees were poor epiphyte hosts on the West Indies while their less densely foliated conspecifics near Rio de Janeiro were good hosts. Moreover, host specificity is expected to depend strongly on the set of locally available tree species. While an epiphyte species may be restricted to a limited number of tree species in a given locality, it may encounter many other tree species with suitable traits within its distributional range—or when extending its distributional range. Thus, it is crucial to bear in mind that findings of host specificity are only valid for the studied location(s).

Although host specificity is usually studied at the species level of both epiphyte and tree, it may also be studied below the species level (Zytynska *et al*. 2011; Whitham *et al*. 2012). For example, host shifts within a species may be due to geographic trait shifts of a tree species as in Mango trees. Moreover, genotypic differences between epiphyte populations or even individuals within populations may translate to differential host specificity below the species level.

The degree of host specificity likely correlates with habitat type. Generalist epiphytes are expected to be correlated with habitats with (i) high host diversity or (ii) high climatic variability. Moreover, a dense mat of non-vascular epiphytes arguably buffers the effect of chemical and physical bark properties (Frei 1973*b*). Thus, host biases should be less pronounced in montane tropical moist forests and lowland temperate rainforests, where bryophyte mats are extremely dense, than in habitats with a high percentage of ‘naked’ bark such as lowland rainforests (Ibisch 1996). Similarly, epiphyte species should show stronger host specificity in habitats where climatic conditions are suboptimal for their performance because the modulating effect of tree traits is stronger under such conditions (Sanford 1974). To summarize, strong host specificity will most likely be associated with exceptionally demanding, aseasonal habitats with a low diversity of potential hosts and a low abundance of non-vascular epiphytes (like rocky outcrop habitats, Table 2).

### Synthesis

**Figure 3.**
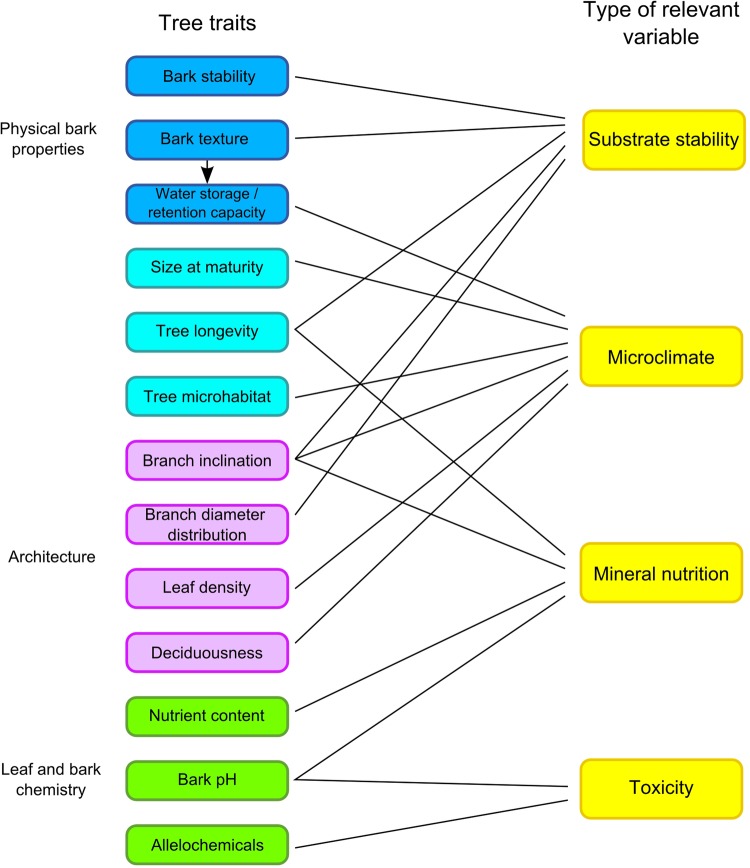
Tree traits related to host specificity and their main influences on four types of variables relevant to epiphyte performance.

A large number of different tree traits have been invoked to cause epiphyte host specificity and certainly more traits could be added to the list. However, this plethora of traits influences a limited number of variables relevant to epiphyte performance: microclimate (including water availability as well as light, humidity and temperature regime), substrate stability, mineral nutrition and toxicity (Fig. 3). Most hypotheses on the mechanisms underlying host specificity involve microclimate and substrate stability and relatively few involve mineral nutrition and toxicity. Correlations between many of the invoked traits and distributional patterns or individual performance of epiphytes have been reported. However, none of the proposed mechanisms has been thoroughly studied, which makes it impossible at this stage to draw conclusions on their relative importance. Since each tree species features a unique combination of trait values it is vital to study a large number of different traits simultaneously. However, so far studies always focused on a very limited number of traits, typically discussing additional traits only when it comes to explain why a particular tree species did not fit into the expected correlation between the considered traits and host specificity. Arguably, many of the listed traits have weak effects on vascular epiphyte performance and none has a paramount effect. Even more complicating, the effect of tree traits will depend on the functional traits of the focal epiphyte species and will be modulated for given epiphyte–tree pairs by local climate and the tree species pool of the study area.

## Outlook

Epiphyte host specificity has received considerable attention as demonstrated by the treatment in >200 publications **[see Supporting Information]**. Unfortunately, in spite of this large number of studies we are still far from a definite understanding of the importance of host tree identity for the structure and dynamics of vascular epiphytes assemblages. In our view, it seems especially rewarding to pursue the following directions: (i) Investigate whether the general finding of widespread structural specificity is corroborated when all possibly confounding factors are considered. (ii) Once patterns are unambiguously documented, study potential mechanisms in order to determine their relative importance. (iii) Test theoretical predictions about the relative strength of host specificity in different habitats and among different groups of dependent flora.

- Since gathering information on tree age and spatial distribution patterns of epiphytes and trees requires a huge effort, we suggest to first explore the influence of these complicating factors in a simulation approach using a range of estimates for the respective parameters. Such an approach could help to decide on minimum sample sizes and appropriate null expectations for the statistical analysis of field data. Moreover, it could be used to assess the error introduced when substrate age is ignored and tree size is used as a proxy for the correlated variables size and age.

As pointed out in the subsection on the investigation of epiphyte host specificity, it is essential to control for tree size, either by standardized sampling or by including it as a covariate in the analysis. Of all size measures, bark surface area should be most closely correlated with epiphyte occupancy and load. The substrate area of a tree species may be determined by summing up the bark surface area over all individuals. Note that we assume for simplicity that substrate quality is independent of substrate type (e.g. trunk and branches) although this is often not true as demonstrated by vertical stratification patterns. Bark surface area can be easily determined if the sampling units are tree trunks only (Wyse and Burns 2011) or if trees lack branches as in tree ferns or palms. Admittedly, it is very labour-intensive to estimate bark surface area of whole trees with a more intricate architecture. A possibility would be to classify tree species by branching type, measure a limited number of trees per class and then model surface area as a function of dbh (see Whittaker and Woodwell 1967). If the response variable is epiphyte abundance per tree, the spatial autocorrelation of epiphytes should be considered when analysing host specificity. Almost two-thirds of the host specificity studies on vascular epiphytes have been performed in tropical or subtropical moist broadleaf forests (Appendix 1 **[see Supporting Information]**). Unfortunately, the high diversity of epiphytes and trees in these forests makes it very difficult to obtain appropriate sample sizes and renders statistical analyses very susceptible to type II errors. Less ambiguous results may be obtained in low diversity systems (e.g. tropical dry or temperate forests)—although the processes shaping epiphyte assemblages may differ substantially between forest types (Burns and Zotz 2010).

Restricted host ranges seem to be exceptional in vascular epiphytes and it is still unclear whether they exist at all. We suggest using the published reports listed in Table 2 as starting points for rigorous quantitative studies with large sample sizes and corroborating conclusions of host-incompatibility based on field observations with seed inoculation and transplantation experiments.

- There are numerous possible mechanisms for host specificity. Some of these have been proposed but have never been tested, for others statistically significant correlations with response variables have been demonstrated. However, the relative importance of these mechanisms is currently unclear.

The suitability of a host species for epiphytes is not caused by its ‘Latin binomial’ (Raffaelli 2007) but rather by the matching of host and epiphyte traits. Thus, shifting the focus from species-associations to correlations of functional traits should advance the understanding of mechanisms and increase the predictive value of studies. Such a functional trait approach would also help to solve the issue of intraspecific tree trait variations due to e.g. ontogenetic changes. Moreover, rare epiphyte and host species that are excluded from analyses of pairwise species-associations for statistical reasons could be considered in correlational analyses of functional traits.

Correlative studies are the first step to identify candidate host traits relevant for host specificity. However, controlled field and laboratory experiments are indispensable to investigate properly the possible effects of bark chemistry and microclimatic variables influenced by host traits. The role of dispersal and of different epiphyte life stages in the determination of host bias patterns should be addressed by seed inoculation and transplantation experiments with plants at different life stages.

- The hypotheses regarding the strength of host specificity in different habitat types and of different groups of dependent flora should be tested with systematic comparative studies as the one by Blick and Burns (2009) on epiphyte, liana and mistletoe networks who found stronger host specificity for the antagonistic networks as compared to the commensalistic one. Host specificity has been linked to rarity in epiphytes (Tremblay *et al*. 1998). A test of the hypothesis that rare species are more host specific than common species would require sampling common and rare species in comparable numbers to ensure comparable statistical certitude for all species—a precondition that is not met by the usual assemblage-based studies.

To conclude, since Schimper's pioneering work, a large body of empirical evidence on host specificity has accumulated and a general pattern of basic host generality and ubiquitous host biases emerges. However, it is now time to place epiphyte host specificity in a broader and more theoretical context. We hope this review inspires new research that helps designing field studies, solving issues regarding the analysis of field data and moving from case studies that document patterns towards a mechanistic understanding of the role of the epiphyte–host interaction for the evolution and ecology of this fascinating group of plants.

## Sources of Funding

This work was supported by the Deutsche Forschungsgemeinschaft (Zo 94/5-1).

## Contributions by the Authors

G.Z. conceived of the manuscript and collected relevant literature over many years. K.W. carried out the literature review, wrote the manuscript and created the figures. G.M.-L., G.Z. and K.W. contributed ideas and edited the manuscript.

## Conflicts of Interest Statement

None declared.

## Supporting Information

The following Supporting Information is available in the online version of this article –

**Table S1.** Lists all studies dealing with host specificity in vascular epiphytes. The methodology of the literature research is described and results based on summary statistics of the table are given.

Additional Information
