# Supplementary material for: Host specificity in vascular epiphytes: a review of methodology, empirical evidence and potential mechanisms
Source: AoB Plants. 2015 Jan 6;7:plu092. doi: 10.1093/aobpla/plu092 (PMC4306756; doi:10.1093/aobpla/plu092)
Supplement: Additional Information [file supp_7_plu092_index.html]

Host specificity in vascular epiphytes: a review of methodology, empirical evidence and potential mechanisms — Additional Information 

# Host specificity in vascular epiphytes: a review of methodology, empirical evidence and potential mechanisms

## Additional Information

Additional Information

**Files in this Data Supplement:**

- Supporting Information - Docx file
