# Supplementary material for: Host specificity in vascular epiphytes: a review of methodology, empirical evidence and potential mechanisms
Source: AoB Plants. 2015 Jan 6;7:plu092. doi: 10.1093/aobpla/plu092 (PMC4306756; doi:10.1093/aobpla/plu092)
Supplement: Additional Information [file supp_plu092_plu092supp.docx]

**SUPPORTING INFORMATION**

**Methods**

We here provide a comprehensive compilation of all publications (issued until April 2014) that deal in some way with host specificity of vascular epiphytes. It includes ecological studies focusing on this subject, or at least discussing it in some detail, as well as anecdotal reports. We searched Web of Science ® (keywords: “host specificity & epiphyt*” and “host preference & epiphyt*”) and relied on the literature database of one of us (GZ), complemented by additional references cited in the revised articles. Primary and secondary literature, grey literature, but also unpublished theses were considered.

**Results**

Starting with Schimper’s (1888) treatise on the Neotropical epiphytic vegetation, Appendix 1 lists 218 publications, of which > 50% were published in the last 15 years (Fig. 1). While we are confident that this list is next to exhaustive for the peer-reviewed ecological literature, we have certainly missed information in species descriptions, floras and unpublished theses.

Most (67%) publications listed in Appendix 1 can be classified as “ecological field studies”. The rest comprises other ecological study types (9%), comments in floras, classifications, species descriptions or species records (7%), anecdotal reports or natural history accounts (11%) and sections of textbooks, monographs or reviews (6%). Of the 184 publications that include some genuine observation on host specificity, 38% are purely observational and another 27% present quantitative data without statistical tests for host specificity. Purely observational accounts of specificity dominate the older literature (Fig. 1), but rather anecdotal comments can also be found in some recent species descriptions (e.g. da Mota *et al.* 2009, Song *et al.* 2009). Although such accounts do not meet modern standards for scientific inference, they contribute to the overall picture (many observations have been made by experienced naturalists) and may serve as starting points for further investigations.

Biogeographically, the Neotropics are best represented (59% of entries) while the indomalayan and afrotropical ecozones are represented by 15% and 12%, respectively. The remaining ecozones (Australasia, Nearctic, Oceania and Palearctic) are each represented by < 10% of the entries. Many entries (41%) are taxonomically unrestricted, i.e. authors investigated whole vascular epiphyte assemblages or discussed the subject without a taxonomic focus. Publications that investigated host specificity of just a few epiphyte species or had a taxonomic focus almost exclusively dealt with three groups (Orchidaceae, Bromeliaceae and Pteridophytes).

**Appendix 1.** Studies dealing with host specificity in vascular epiphytes

| Reference | Biome | Ecozone | Epiphyte group | Quality | Comments |
| --- | --- | --- | --- | --- | --- |
| *Publication type: Ecological field study* | |  |  |  |  |
| Ackerman *et al.* (1989) | 02 | NT | OR | quantitative |  |
| Ackerman *et al.* (1996) | 01 | NT | OR | statistics |  |
| Addo-Fordjour *et al.* (2009) | 01 ^u^ | AT | VA | statistics | botanical garden |
| Adhikari *et al.* (2012) | 01 ^u^ | IM | OR | quantitative |  |
| Aguirre *et al.* (2010) | 02 | NT | VA | statistics |  |
| Alemayehu (2006) | 01 | AT | VA | quantitative | MSc thesis |
| Andrade and Nobel (1996) | 01 | NT | CA | statistics |  |
| Andrade and Nobel (1997) | 01 | NT | CA, PT | statistics |  |
| Annaselvam and Parthasarathy (2001) | 01 | IM | VA | quantitative |  |
| Azemi *et al.* (1996) | 01 | IM | VA | statistics |  |
| Benavides *et al.* (2011) | 01 | NT | VA | statistics |  |
| Bennett (1984) | 01 | NT | BR | statistics |  |
| Bennett (1987) | 01 | NT | BR | statistics |  |
| Benzing and Renfrow (1974) | 01 | NT | BR, OR | own obs | mentions host specificity in discussion |
| Benzing (1978) | 01 | NT | BR | quantitative | seed and seedling transplantation experiments |
| Benzing (1981) | 01 | NT | BR | own obs | mentions host specificity in discussion |
| Bergstrom and Carter (2008) | 01 | NA | OR | quantitative |  |
| Bernal *et al.* (2005) | 07 | NT | BR | statistics |  |
| Bittner and Trejos-Zelaya (1997) | 01 | NT | VA | statistics |  |
| Blick and Burns (2009) | 04 | AA | VA | statistics |  |
| Boelter *et al.* (2011) | 01 ^p^ | NT | VA | statistics | Araucaria forest and exotic tree plantations |
| Boelter *et al.* (2014) | 01 | NT | VA | (statistics) | tests for assoc. with bark type |
| Bonnet *et al.* (2007) | 01 | NT | BR | (statistics) | correlations of epiphytes with host traits |
| Boyer (1964) | 01, 02 | AT | PT | own obs |  |
| Breier (2005) | 01 | NT | VA | statistics | PhD thesis |
| Brown (1990) | 01 | NT | VA | quantitative |  |
| Burns and Dawson (2005) | 04 | AA | VA | statistics |  |
| Burns (2007) | 04 | AA | VA | statistics |  |
| Burns and Zotz (2010) | 01 | NT | VA | statistics |  |
| Buzatto *et al.* (2008) | 01 | NT | VA | quantitative |  |
| Cach-Pérez *et al.* (2013) | 01, 02, 14 | NT | BR | quantitative |  |
| Caldiz *et al.* (1993) | 01 ^u^ | NT | BR | (statistics) |  |
| Callaway *et al.* (2001) | 01 | NA | BR | statistics |  |
| Callaway *et al.* (2002) | 01 | NA | BR, PT | statistics |  |
| Cardelús and Chazdon (2005) | 01 | NT | VA | secondary | mentions host specificity in discussion |
| Cardelús (2007) | 01 | NT | VA | statistics |  |
| Carlsen (2000) | 01 | NT | VA | quantitative |  |
| Castaño-Meneses *et al.* (2003) | 04 | NA | BR | statistics |  |
| Castro-Hernández *et al.* (1999) | 01, 03 | NT | BR | own obs |  |
| Chang (2005) | 04 | IM | VA | quantitative | MSc thesis, in Chinese, abstract in English |
| Chauhan *et al.* (2003) | 01 | IM | PT | own obs |  |
| Chomba *et al.* (2011) | 07 | AT | VA | (statistics) |  |
| Clements (1987) | 01 | AA | OR | own obs | conference publ. |
| Crain and Tremblay (2012) | 01 | NT | OR | quantitative |  |
| de C. Guaraldo *et al.* (2013) | 01 | NT | CA | statistics |  |
| de la Rosa Manzano *et al.* (2014) | 02 | NT | OR | quantitative |  |
| Dejean *et al.* (1995) | 01 | NT | BR, OR | statistics |  |
| Dejean and Olmsted (1997) | 01 | NT | BR | quantitative |  |
| del Carmen Zanetti Berrocal (2004) | 04 | NT | VA | quantitative | thesis (Ingenerio forestal) |
| Diaz Santos (2000) | 01 | NT | OR | statistics |  |
| Einzmann *et al.* (2014) | 01 | NT | VA | statistics |  |
| Ek *et al.* (1997) | 01 | NT | VA | own obs |  |
| Flores-Palacios and Garcia-Franco (2008) | 01 | NT | VA | own obs |  |
| Fontoura (1995) | 01 | NT | BR | statistics |  |
| Fontoura *et al.* (2009) | 02 | NT | VA | quantitative |  |
| Frei (1973b) | 01 | NT | OR | own obs |  |
| García-Franco and Peters (1987) | 04 | NT | BR | statistics |  |
| García-González *et al.* (2011) | 01 ^p^ | NT | OR | (statistics) |  |
| García-Suárez *et al.* (2003) | 07 | NT | BR | quantitative |  |
| García-Suárez *et al.* (2006) | 07 | NT | BR | quantitative | same results as in García-Suárez (2003) |
| Garth (1964) | 04 | NA | BR | own obs |  |
| Geraldino *et al.* (2010) | 01 | NT | VA | quantitative |  |
| Gottsberger and Morawetz (1993) | 07 | NT | VA | quantitative |  |
| Gowland *et al.* (2011) | 04 | AA | OR | statistics |  |
| Gowland *et al.* (2013) | 04 | AA | OR | secondary | orchid mycorrhiza as mechanism for host specificity |
| Haro-Carrión (2004) | 01 | NT | BR | (statistics) |  |
| Hassall and Kirkpatrick (1985) | 01 | OC | VA | quantitative |  |
| Henderson (1935) | 01 | IM | OR | own obs |  |
| Hernández-Rosas and Carlsen (2003) | 01 | NT | VA | own obs |  |
| Hietz and Hietz-Seifert (1995b) | 01 | NT | VA | statistics |  |
| Hietz and Hietz-Seifert (1995a) | 01, 02, 03 | NT | VA | statistics |  |
| Hirata *et al.* (2009) | 04 | PA | VA | statistics |  |
| Husk *et al.* (2004) | 04 | NA | BR | secondary | mineral composition of bromeliad on different hosts |
| Ipulet and Kasenene (2008) | 01, 02, 07 ^p^ | AT | MO | quantitative |  |
| Janzen (1969) | 01 | NT | VA | own obs |  |
| Johansson (1974) | 01 | AT | VA | own obs |  |
| Johnson and Awan (1972) | 01 ^u^ | IM | VA | quantitative | study performed in urban area |
| Kaufmann *et al.* (2001) | 01 | IM | VA | secondary | hypothesis on ants mediating host specificity |
| Kersten *et al.* (2009) | 01 | NT | VA | quantitative |  |
| Kiew and Anthonysamy (1987) | 01 | IM | VA | quantitative |  |
| Köster *et al.* (2011) | 01 | NT | VA | statistics |  |
| Kundu and Pal (1998) | 02 ^u^ | IM | PT | own obs | botanical garden |
| Laube and Zotz (2006) | 01 | NT | VA | statistics |  |
| López-Villalobos *et al.* (2008) | 07 | NT | BR | secondary | studies bark peeling as avoidance mechanism |
| Malizia (2003) | 01 | NT | VA | statistics |  |
| Martin *et al.* (2007) | 01 | IM | OR | statistics | compares epiphyte physiological parameters |
| Martínez-Meléndez *et al.* (2008) | 01 | NT | VA | statistics |  |
| Matias *et al.* (1996) | 07 | NT | OR | secondary | mentions host specificity in discussion |
| Medeiros *et al.* (1993) | 01 | OC | VA | statistics |  |
| Medeiros and Gonçalves Jardim (2011) | 01 | NT | OR | quantitative |  |
| Mehltreter *et al.* (2005) | 01 | NT | VA | statistics |  |
| Merwin *et al.* (2003) | 01 | NT | BR | statistics |  |
| Migenis and Ackerman (1993) | 01 | NT | OR | quantitative |  |
| Mondragón *et al.* (1999) | 02 | NT | BR | own obs | mentions host specificity in methods |
| Mondragón *et al.* (2004) | 02 | NT | BR | own obs | mentions host specificity in methods |
| Moran *et al.* (2003) | 01 | NT | PT | statistics |  |
| Moran and Russell (2004) | 01 | NT | PT | statistics |  |
| Mucunguzi (2008) | 01 | AT | OR | quantitative |  |
| Muñoz *et al.* (2003) | 04 | NT | VA | statistics |  |
| Mursidawati *et al.* (1999) | 01 | IM | OR | quantitative |  |
| Nieder *et al.* (2000) | 01 | NT | VA | quantitative |  |
| Noumi *et al.* (2010) | 01 | AT | VA | quantitative |  |
| Obermüller *et al.* (2012) | 01 | NT | VA | quantitative |  |
| Otero *et al.* (2007) | 02 | NT | OR | statistics |  |
| Pessin (1925) | 04 | NA | PT | own obs |  |
| Piazzon *et al.* (2011) | 04 | NT | VA | statistics | network approach, focus not on host specificity |
| Raventós *et al.* (2011) | 02 | NT | OR | secondary | mentions host specificity in discussion |
| Reif and Allen (1988) | 04 | AA | VA | own obs |  |
| Reyes-García *et al.* (2008) | 02 | NT | BR | statistics | correlations of epiphytes with host traits |
| Riveros and Ramírez (1978) | 04 | NT | VA | quantitative |  |
| Roberts *et al.* (2005) | 04 | AA | PT | statistics |  |
| Sanford (1968) | 01 | AT | OR | secondary |  |
| Sáyago *et al.* (2013) | 02 | NT | BR | statistics | network approach |
| Schlesinger and Marks (1977) | 04 | NA | BR | own obs |  |
| Schmitt and Windisch (2005) | 01 | NT | VA | secondary | mentions host specificity in discussion |
| Schmitt and Windisch (2010) | 01, 02 | NT | VA | secondary | mentions host specificity in discussion |
| Scott and Rowley (1975) | 04 | AA | VA | quantitative |  |
| Sharma (2010) | 04 | IM | OR | quantitative | MSc thesis |
| Silva *et al.* (2010) | 02 | NT | OR | statistics | network approach |
| Sonké *et al.* (2001) | n/a ^p^ | AT | VA | own obs | hedge vegetation in village |
| Sosa and Platas (1998) | 01, 02, 03 ^p^ | NT | OR | own obs |  |
| Soto Arenas (1994) | 02 | NT | OR | own obs | conference publ. |
| ter Steege and Cornelissen (1989) | 01 | NT | VA | statistics |  |
| Tomazini (2007) | 04 | NT | VA | quantitative | PhD thesis |
| Trapnell and Hamrick (2006) | 01 | NT | OR | quantitative | semiquantitative |
| Traxmandlová *et al.* (2012) | 01 | IM | OR | (statistics) |  |
| Tremblay *et al.* (Tremblay *et al.* 1998) | 01 | NT | OR | quantitative |  |
| Valdivia (1977) | 01 | NT | VA | quantitative |  |
| Valencia-Díaz *et al.* (2014) | 02 | NT | BR | secondary | mentions host specificity in discussion |
| Válka Alves *et al.* (2008) | 07 | NT | VA | own obs |  |
| Vergara-Torres *et al.* (2010) | 02 | NT | VA | statistics |  |
| Waechter and Baptista (2004) | 01 | NT | OR | quantitative |  |
| Wallace (1981) | 01, 02 | AA | VA | quantitative | PhD thesis |
| Watthana (2004) | 01 | IM | OR | quantitative |  |
| Watthana and Pedersen (2008) | 01, 02 | IM | OR | quantitative |  |
| Wee (1978) | 01 ^u^ | IM | VA | quantitative |  |
| Went (1940) | 01 | IM | VA | quantitative |  |
| Wolf (1994) | 01 | NT | VA | statistics |  |
| Wyse and Burns (2011) | 04 | AA | VA | statistics |  |
| Xu and Liu (2005) | 01 | IM | VA | quantitative | in Chinese, abstract in English |
| Zapfack *et al.* (1996) | 01 | AT | PT | (statistics) |  |
| Zhang *et al.* (2010) | 01 | IM | PT | statistics |  |
| Zimmerman and Olmsted (1992) | 02 | NT | BR, OR | quantitative |  |
| Zotz *et al.* (1999) | 01 | NT | VA | own obs | mentions host specificity in discussion |
| Zotz (2002) | 04 | PA | PT | quantitative |  |
| Zotz and Vollrath (2003) | 01 | NT | VA | secondary | mentions host specificity in discussion |
| Zotz and Schultz (2008) | 01 | NT | VA | statistics |  |
| Zotz *et al.* (2014) | 01 | NT | VA | statistics |  |
|  |  |  |  |  |  |
| *Publication type: other study type (e.g. floristic survey, biogeographic study, laboratory experiment)* | | | | | |
| Braithwaite (1986) | 01 | OC | PT | own obs |  |
| Cortez (2001) | 01 | NT | PT | own obs |  |
| Dagar and Jeyamurthy (1990) | 01 | IM | VA | own obs |  |
| de Castro Ribeiro and de Paula (2009) | 07 | NT | BR | own obs | conclusions based on only 3 individuals |
| Droissart *et al.* (2011) | 01, 07 | AT | OR | secondary | mentions host specificity in discussion |
| Frei and Dodson (1972) | 01 | NT | OR | secondary | effect of bark extract on orchid development |
| Frei (1973a) | 01 | NT | OR | secondary | effect of bark extract on orchid development |
| Gonzalez-Astorga *et al.* (2004) | 02 | NT | BR | secondary | mentions host specificity in discussion |
| Harshani *et al.* (2014) | 01 | IM | OR | (statistics) | tests allelopathy of bark extracts on germination |
| Hedge (1985) | 01, 02, 03, 04 | IM | OR | own obs |  |
| Hemp (2001) | 01 | AT | PT | quantitative |  |
| Ibisch (1996) | 01 | NT | VA | quantitative | PhD thesis |
| Jarman *et al.* (1986) | 04 | AA | PT | own obs |  |
| Kolbek (1995) | 04 | PA | VA | own obs | methods not described |
| Mesler (1975) | 01 | NT | PT | own obs |  |
| Morris (1968) | 07 | AT | OR | own obs |  |
| Morris (1970) | n/a | AT | OR | own obs |  |
| Porembski (1996) | 07 | AT | OR | own obs |  |
| Sehgal and Mehra (1984) | 01, 03 | IM | OR | own obs |  |
| Valencia-Díaz *et al.* (2010) | 02 | NT | BR | secondary | tests mechanism (allelochemicals in bark) |
|  |  |  |  |  |  |
|  |  |  |  |  |  |
| *Publication type: flora, classification, species description, species record* | | | | |  |
| Cribb *et al.* (2002) | 01 ^p^ | AT | OR | own obs |  |
| da Mota *et al.* (2009) | 07 | NT | OR | own obs |  |
| Hernández and Diaz (2000) | 01 | NT | OR | own obs |  |
| Ibisch *et al.* (1995) | 01 | NT | PT | own obs |  |
| La Croix *et al.* (1991) | n/a | AT | OR | secondary |  |
| Li and Dao (2014) | 04, 05 | PA | OR | own obs |  |
| Mayo *et al.* (2000) | 01, 07 | NT | AR | own obs | assoc. of aroids with terrestrial bromeliads |
| Mickel and Beitel (1988) | n/a | NT | PT | secondary | positive association of some species with tree ferns |
| Moran and Riba (1995) | n/a | NT | PT | secondary | positive association of some species with tree ferns |
| Piers (1968) | n/a | AT | OR | own obs |  |
| Pridgeon (2005) | 02 | NT | OR | own obs |  |
| Proctor (1989) | n/a | NT | PT | own obs | one species especially found on tree ferns |
| Rupp and McGillivray (1969) | n/a | AA | OR | own obs |  |
| Song *et al.* (2009) | 01 | IM | OR | own obs |  |
| Van den Berg *et al.* (2006) | 07 | NT | OR | own obs |  |
|  |  |  |  |  |  |
| *Publication type: natural history, anecdotal* | |  |  |  |  |
| Allen (1959) | n/a | NT | OR | own obs | cultivators journal |
| Bir (1989) | 01 | IM | PT | secondary | conference publ.; origin of conclusions? |
| Birge (1911) | 04 | NA | BR | own obs |  |
| Copeland (1916) | n/a | IM | PT | own obs |  |
| Curtis (1947) | 02 | NT | OR | own obs |  |
| Dickinson (1968) | 02 | NT | OR | own obs | cultivators journal |
| Diem and De Lichtenstein (1959) | 04 | NT | PT | own obs |  |
| Dudgeon (1923) | 02 | IM | VA | own obs |  |
| Eggeling (1947) | 01 | AT | VA | own obs |  |
| Fraser and Vickery (1938) | 01 | AA | VA | own obs |  |
| Herndon (1996) | n/a | AT | OR | secondary | cultivators journal; repeats Lecoufle (1964) |
| Kennedy (1972) | n/a | AT | OR | secondary | cultivators journal; repeats Lecoufle (1964) |
| Lecoufle (1964) | 01 | AT | OR | own obs | cultivators journal |
| Neger (1913) | n/a | n/a | VA | secondary |  |
| Nir (1988) | 01 ^p^ | NT | OR | own obs | cultivators journal, coffee plantation |
| Oliver (1930) | n/a | AA | VA | own obs |  |
| Pollard (1973) | 01 | NT | OR | own obs | cultivators journal |
| Porembski (2003) | 07 | AT, NT | OR | own obs |  |
| Reiche (1907) | n/a | NT | VA | own obs |  |
| Schimper (1888) | n/a | NT | VA | own obs |  |
| Sulit (1950) | n/a | IM | OR | own obs | cultivators journal |
| Sulit (1953) | n/a | IM | OR | own obs | cultivators journal |
| Van Oye (1924) | 01 | IM | VA | own obs |  |
|  |  |  |  |  |  |
| *Publication type: textbook, review, monograph* | | |  |  |  |
| Arditti (1992) | n/a | n/a | OR | secondary |  |
| Bartels and Chen (2012) | n/a | n/a | VA | secondary | mentions host specificity in discussion |
| Benzing (1990) | n/a | n/a | VA | secondary |  |
| Benzing (1995) | n/a | n/a | VA | secondary |  |
| Dressler (1981) | n/a | n/a | OR | secondary |  |
| Fontoura *et al.* (1997) | 01 | NT | VA | secondary |  |
| Ghazoul and Sheil (2010) | 01 | n/a | VA | secondary | mentions host specificity in discussion |
| Mehltreter (2008) | n/a | n/a | PT | quantitative |  |
| Nieder *et al.* (2001) | n/a | n/a | VA | secondary |  |
| Sanford (1974) | n/a | n/a | OR | own obs |  |
| Schnell (1970) | n/a | n/a | VA | secondary |  |
| Sehnem (1977) | n/a | NT | PT | own obs |  |
| Zotz (2005) | n/a | n/a | VA | secondary | mentions host specificity in discussion |

Abbreviations: n/a = not available

- Ecozone: notes in which ecozone (Olson *et al.* 2001) study has been performed. Categories: Australasia (AA), Afrotropic (AT), Indomalaya (IM), Nearctic (NA), Neotropic (NT), Oceania (OC), Palearctic (PA).
- Biome: notes to which biome (Olson *et al.* 2001) publication refers; multiple entries are caused by sites in different biomes or large reference area. Categories: Tropical and subtropical moist broadleaf forests (01), Tropical and subtropical dry broadleaf forests (02), Tropical and subtropical coniferous forests (03), Temperate broadleaf and mixed forests (04), Temperate coniferous forest (05), Tropical and subtropical grasslands, savannas, and shrublands (07), Mangrove (14).
- Epiphyte group: notes to which taxonomic group of vascular epiphytes publication refers. Categories: Araceae (AR), Bromeliaceae (BR), Cactaceae (CA), Moraceae (MO), Orchidaceae (OR), Pteridophytes (PT), publication refers to all vascular epiphytes in studied assemblage or in general (VA).
- Quality: notes publication quality. Categories: unknown (n/a), conclusions not based on own observations (secondary), conclusions based on own observations presented in the paper (own obs), conclusions based on quantitative data (quantitative), conclusions based on statistical test (statistics).

^u^ Study at least partly performed in urban area or botanical garden.

^p^ Study at least partly performed in plantation.

**LITERATURE CITED**

Ackerman JD, Montalvo AM, Vera AM. 1989. Epiphyte host specificity of *Encyclia krugii*, a Puerto Rican endemic orchid. *Lindleyana* 4:74-77.

Ackerman JD, Sabat A, Zimmerman JK. 1996. Seedling establishment in an epiphytic orchid: an experimental study of seed limitation. *Oecologia* 106:192-198.

Addo-Fordjour P, Anning AK, Addo MG, Osei MF. 2009. Composition and distribution of vascular epiphytes in a tropical semideciduous forest, Ghana. *African Journal of Ecology* 47:767-773.

Adhikari YP, Fischer HS, Fischer A. 2012. Host tree utilization by epiphytic orchids in different land-use intensities in Kathmandu Valley, Nepal. *Plant Ecology* 213:1393-1412.

Aguirre A, Guevara R, García M, López JC. 2010. Fate of epiphytes on phorophytes with different architectural characteristics along the perturbation gradient of *Sabal mexicana* forests in Veracruz, Mexico. *Journal of Vegetation Science* 21:6-15.

Alemayehu T. 2006. *Diversity and ecology of vascular epiphytes in Harenna afromontane forest, Bale, Ethiopia,* MSc Thesis, Addis Ababa University, Ethiopia.

Allen PH. 1959. Orchid hosts in the tropics. *American Orchid Society Bulletin* 28:243-244.

Andrade JL, Nobel PS. 1996. Habitat, CO_2_ uptake, and growth for the CAM epiphytic cactus *Epiphyllum phyllanthus* in a Panamanian tropical forest. *Journal of Tropical Ecology* 12:291-306.

Andrade JL, Nobel PS. 1997. Microhabitats and water relations of epiphytic cacti and ferns in a lowland neotropical forest. *Biotropica* 29:261-270.

Annaselvam J, Parthasarathy N. 2001. Diversity and distribution of herbaceous vascular epiphytes in a tropical evergreen forest at Varagalaiar, Western Ghats, India. *Biodiversity and Conservation* 10:317-329.

Arditti J. 1992. *Fundamentals of orchid biology,* New York: Wiley-Liss, Inc.

Azemi H, Laman TG, Budhi S. 1996. Distribution and abundance of vascular epiphytes and hemiepiphytic *Ficus* on dipterocarps in Gunung Palung National Park, West Kalimantan, Indonesia. *Tropical Biodiversity* 3:181-192.

Bartels SF, Chen HYH. 2012. Mechanisms regulating epiphytic plant diversity. *Critical Reviews in Plant Sciences* 31:391-400.

Benavides AM, Vasco A, Duque AJ, Duivenvoorden JF. 2011. Association of vascular epiphytes with landscape units and phorophytes in humid lowland forests of Colombian Amazonia. *Journal of Tropical Ecology* 27:223-237.

Bennett BC. 1984. A comparison of the spatial distribution of *Tillandsia flexuosa* and *T. pruinosa*. *Florida Scientist* 47:141-144.

Bennett BC. 1987. Spatial distribution of *Catopsis* and *Guzmania* (Bromeliaceae) in southern Florida. *Bulletin of the Torrey Botanical Club* 114:265-271.

Benzing DH. 1978. Germination and early establishment of *Tillandsia circinnata* Schlecht. (Bromeliaceae) on some of its hosts and other supports in Southern Florida. *Selbyana* 5:95-106.

Benzing DH. 1981. The population dynamics of *Tillandsia circinnata* (Bromeliaceae): cypress crown colonies in Southern Florida. *Selbyana* 5:256-263.

Benzing DH. 1990. *Vascular epiphytes. General biology and related biota,* Cambridge: Cambridge University Press.

Benzing DH. 1995. Vascular epiphytes. In: Lowman MD, Nadkarni NM eds. *Forest Canopies.* 1 edn. San Diego: Academic Press, 225-254

Benzing DH, Renfrow A. 1974. The nutritional status of *Encyclia tampense* and *Tillandsia circinnata* on *Taxodium ascendens* and the availability of nutrients to epiphytes on this host in South Florida. *Bulletin of the Torrey Botanical Club* 101:191-197.

Bergstrom BJ, Carter R. 2008. Host-tree selection by an epiphytic orchid, *Epidendrum magnoliae* Muhl. (Green Fly Orchid), in an inland hardwood hammock in Georgia. *Southeastern Naturalist* 7:571-580.

Bernal R, Valverde T, Hernandez-Rosas L. 2005. Habitat preference of the epiphyte *Tillandsia recurvata* (Bromeliaceae) in a semi-desert environment in Central Mexico. *Canadian Journal of Botany* 83:1238-1247.

Bir SS. 1989. Evolutionary trends in the pteridophytic flora of India. In: Bir SS, Saggoo MIS eds. *Botany Section 75th Indian Science Congress Pune.* Pune, Today & Tomorrow's Printers and Publishers, New Delhi.

Birge WI. 1911. *The anatomy and some biological aspects of "ball moss", Tillandsia recurvata L.,* Austin, Texas: Bulletin of the University of Texas.

Bittner J, Trejos-Zelaya J. 1997. Analysis of the vascular epiphytes of tree ferns in a montane rain forest in Costa Rica. *Revista de Matemática: Teoría y Aplicaciones* 4:63-73.

Blick R, Burns KC. 2009. Network properties of arboreal plants: Are epiphytes, mistletoes and lianas structured similarly? *Perspectives in Plant Ecology Evolution and Systematics* 11:41-52.

Boelter CR, Dambros CS, Nascimento HEM, Zartman CE. 2014. A tangled web in tropical tree-tops: effects of edaphic variation, neighbourhood phorophyte composition and bark characteristics on epiphytes in a central Amazonian forest. *Journal of Vegetation Science* 25:1090-1099.

Boelter CR, Zartman CE, Fonseca CR. 2011. Exotic tree monocultures play a limited role in the conservation of Atlantic Forest epiphytes. *Biodiversity and Conservation* 20:1255-1272.

Bonnet A, Queiroz MHd, Lavoranti OJ. 2007. Relações de bromélias epifíticas com características dos forófitos em diferentes estádios sucessionais da floresta ombrófila densa, Santa Catarina, Brasil. *Floresta* 37:83-94.

Boyer Y. 1964. Contribution a l'étude de l'écophysiologie de deux fougères épiphytes: *Platycerum stemari*a (Beauv.) Desv. et *P. angolense* Welch. *Annales des Sciences Naturelles, Botanique, Paris* 12:87-228.

Braithwaite AF. 1986. *Tmesipteris* in Vanuatu (New Hebrides). *Fern Gazette* 13:87-96.

Breier TB. 2005. *O epifitismo vascular em florestas do sudeste do Brasil,* PhD Thesis, Universidade Estadual de Campinas, Brazil.

Brown AD. 1990. El epifitismo en las selvas montanas del Parque Nacional "El Rey", Argentina: composición florística y patrón de distribución. *Revista de Biología Tropical* 38:155-166.

Burns KC. 2007. Network properties of an epiphyte metacommunity. *Journal of Ecology* 95:1142-1151.

Burns KC, Dawson J. 2005. Patterns in the diversity and distribution of epiphytes and vines in a New Zealand forest. *Austral Ecology* 30:891-899.

Burns KC, Zotz G. 2010. A hierarchical framework for investigating epiphyte assemblages: networks, metacommunities and scale. *Ecology* 377-385.

Buzatto CR, Severo BMA, Waechter JL. 2008. Composição florística e distribuição ecológica de epífitos vasculares na Floresta Nacional de Passo Fundo, Rio Grande do Sul. *Iheringia Serie Botanica* 63:231-239.

Cach-Pérez MJ, Andrade JL, Chilpa-Galván N, Tamayo-Chim M, Orellana R, Reyes-García C. 2013. Climatic and structural factors influencing epiphytic bromeliad community assemblage along a gradient of water-limited environments in the Yucatan Peninsula, Mexico. *Tropical Coservation Science* 6:283-302.

Caldiz DO, Beltrano J, Fernández LV, Andía I. 1993. Survey of *Tillandsia recurvata* L.: preference, abundance and its significance for natural forests. *Forest Ecology and Management* 57:161-168.

Callaway RM, Reinhart KO, Moore GW, Moore DJM, Pennings SC. 2002. Epiphyte host preferences and host traits: mechanisms for species-specific interactions. *Oecologia* 132:221-230.

Callaway RM, Reinhart KO, Tucker SC, Pennings SC. 2001. Effects of epiphytic lichens on host preference of the vascular epiphyte *Tillandsia usneoides*. *Oikos* 94:433-441.

Cardelús CL. 2007. Vascular epiphyte communities in the inner-crown of *Hyeronima alchorneoides* and *Lecythis ampla* at La Selva Biological Station, Costa Rica. *Biotropica* 39:171-176.

Cardelús CL, Chazdon RL. 2005. Inner-crown microenvironments of two emergent tree species in a lowland wet forest. *Biotropica* 37:238-244.

Carlsen M. 2000. Structure and diversity of the vascular epiphyte community in the overstory of a tropical rain forest in Surumoni, Amazonas State, Venezuela. *Selbyana* 21:7-10.

Castaño-Meneses G, García-Franco JG, Palacios-Vargas JG. 2003. Spatial distribution patterns of *Tillandsia violacea* (Bromeliaceae) and support trees in an altitudinal gradient from a temperate forest in Central Mexico. *Selbyana* 24:71-77.

Castro-Hernández JC, Wolf JHD, Garcia-Franco JG, González-Espinosa M. 1999. The influence of humidity, nutrients and light on the establishment of the epiphytic bromeliad *Tillandsia guatemalensis* in the highlands of Chiapas, Mexico *Revísta de Biologia Tropical* 47:763-773.

Chang M. 2005. *The vines and epiphytes of a warm-temperate evergreen broad-leaved forest in Chungtzekuan area,* MSc MSc Thesis, National University of Tainan, Taiwan.

Chauhan N, Padalia H, Gupta S, Porwal MC, Roy PS. 2003. *Psilotum complanatum* Sw., a rare epiphytic fern ally of Great Nicobar Island: Exploration and habitat monitoring. *Current Science* 85:193-197.

Chomba C, Senzota R, Chabwela H, Nyirenda V. 2011. The influence of host tree morphology and stem size on epiphyte biomass distribution in Lusenga Plains National Park, Zambia. *Journal of Ecology and the Natural Environment* 3:370-380.

Clements MA. 1987. Orchid-fungus-host associations of epiphytic orchids. In: Saito K, Tanaka R eds. *Proceedings of the 12th World Orchid Conference.* Tokyo.

Copeland EB. 1916. Natural selection and the dispersal of species. *The Philippine Journal of Science* 6:147-171.

Cortez L. 2001. Epiphytic pteridophytes found in the Cyatheaceae and Dicksoniaceae from cloud forests of Venezuela. *Gayana Botanica* 58:13-23.

Crain BJ, Tremblay RL. 2012. Update on the distribution of *Lepanthes caritensis*, a rare Puerto Rican endemic orchid. *Endangered Species Research* 18:89-94.

Cribb PJ, Du Puy D, Bosser J. 2002. An unusual new epiphytic species of *Eulophia* (Orchidaceae) from southeastern Madagascar. *Adansonia* 24:169-172.

Curtis JT. 1947. Ecological observations on the orchids of Haiti. *American Orchid Society Bulletin*:263-269.

da Mota RC, de Barros F, Stehmann JR. 2009. Two new species of Orchidaceae from Brazil: *Bulbophyllum carassense* and *Lepanthopsis vellozicola*. *Novon* 19:380-387.

Dagar JC, Jeyamurthy A. 1990. Ordination of dependent synusiae in tropical rain forests of South Andaman with special reference to host trees. *Indian Forester* 116:381-389.

de C. Guaraldo A, de O. Boeni B, Pizo MA. 2013. Specialized seed dispersal in epiphytic cacti and convergence with mistletoes. *Biotropica* 45:465-473.

de Castro Ribeiro OB, de Paula CC. 2009. Bromeliads in the extreme south of the Espinhaço Range, Minas Gerais, Brazil. *Selbyana* 30:107-113.

de la Rosa Manzano E, Andrade JL, Zotz G, Reyes-García C. 2014. Epiphytic orchids in tropical dry forests of Yucatan, Mexico - Species occurrence, abundance and correlations with host tree characteristics and environmental conditions. *Flora* 209:100-109.

Dejean A, Olmsted IC. 1997. Ecological studies on *Aechmea bracteata* (Swartz) (Bromeliaceae). *Journal of Natural History* 31:1313-1334.

Dejean A, Olmsted IC, Snelling RR. 1995. Tree-epiphyte-ant relationships in the low inundated forest of Sian Ka'an Biospere reserve, Quintana Roo, Mexico. *Biotropica* 27:57-70.

del Carmen Zanetti Berrocal S. 2004. *Estudio de la vegetación epífita vascular en un bosque adulto de olivillo (Temuco, Chile).* Ingeniero Forestal Ingeniero Forestal Thesis, Universidad de la Frontera, Temuco.

Diaz Santos F. 2000. Orchid preference for host tree genera in a Nicaraguan tropical rain forest. *Selbyana* 21:25-29.

Dickinson S. 1968. Mexico's "Lirio or Flor de Mayo" - *Laelia speciosa*. *American Orchid Society Bulletin* 37:1062-1064.

Diem J, De Lichtenstein JS. 1959. Las Himenofiláceas del área argentino-chilena del sud. *Darwiniana* 11:611-760.

Dressler RL. 1981. *The orchids. Natural history and classification,* Cambridge: Harvard University Press.

Droissart V, Sonke B, Hardy OJ, Simo M, Taedoumg H, Nguembou CK, Stevart T. 2011. Do plant families with contrasting functional traits show similar patterns of endemism? A case study with Central African Orchidaceae and Rubiaceae. *Biodiversity and Conservation* 20:1507-1531.

Dudgeon W. 1923. Succession of epiphytes in the *Quercus incana* forest at Landour, western Himalayas. Preliminary notes. *Journal of the Indian Botanical Society* 3:270-272.

Eggeling WJ. 1947. Observations on the ecology of the Budongo Forest, Uganda. *Journal of Ecology* 34:20-87.

Einzmann HJR, Beyschlag J, Hofhansl F, Wanek W, Zotz G. 2014. Host tree phenology affects vascular epiphytes at the physiological, demographic and community level. *AoB Plants*.

Ek RC, Ter Steege H, Biesmeijer KC. 1997. Vertical distribution and associations of vascular epiphytes in four different forest types in the Guianas. In: Tropenbos ed. *Botanical diversity in the tropical rain forest of Guyana.* Utrecht: Tropenbos, 65-89

Flores-Palacios A, Garcia-Franco JG. 2008. Habitat isolation changes the beta diversity of the vascular epiphyte community in lower montane forest, Veracruz, Mexico. *Biodiversity and Conservation* 17:191-207.

Fontoura T. 1995. Distribution patterns of five Bromeliaceae genera in Atlantic rainforest, Río de Janeiro State, Brazil. *Selbyana* 16:79-93.

Fontoura T, Rocca MA, Schilling AC, Reinert F. 2009. Epífitas da floresta seca da Reserva Ecológica Estadual de Jacarepiá, sudeste do Brasil: relações com a comunidade arbórea. *Rodriguésia* 60:171-185.

Fontoura T, Sylvestre LS, Vaz AMS, Vieira CM. 1997. Epífitas vasculares, hemiepífitas e hemiparasitas da Reserva Ecológica de Macaé de Cima. In: Lima HC, Guedes-Bruni RR eds. *Serra de Macaé de Cima: diversidade florística e conservação da Mata Atlantica.* Rio de Janeiro: Editora do Jardim Botânico do Rio de Janeiro, 89-101

Fraser L, Vickery JW. 1938. The ecology of the upper the Williams River and Barrington Tops districts. II. The rain-forest formations. *Proceedings of the Linnean Society of New South Wales* 63:139-184.

Frei JK. 1973a. Effect of bark substrate on germination and early growth of *Encyclia tampensis* seeds. *American Orchid Society Bulletin* 42:701-708.

Frei JK. 1973b. Orchid ecology in a cloud forest in the mountains of Oaxaca, Mexico. *American Orchid Society Bulletin* 42:307-314.

Frei JK, Dodson CH. 1972. The chemical effect of certain bark substrates on the germination and early growth of epiphytic orchids. *Bulletin of the Torrey Botanical Club* 99:301-307.

García-Franco JG, Peters CM. 1987. Patrón espacial y abundancia de *Tillandsia spp.* a través de un gradiente altitudinal en los altos de Chiapas, México. *Brenesia* 27:35-45.

García-González A, Damon A, Esparza Olguín LG, Valle-Mora J. 2011. Population structure of *Oncidium poikilostalix* (Orchidaceae), in coffee plantations in Soconusco, Chiapas, México *Lankesteriana* 11:21-32.

García-Suárez MD, Rico-Gray V, Molina-Aceves N, Serrano H. 2006. In-vitro germination and clonal propagation of endemic *Tillandsia califanii* Rauh (Bromeliaceae) from Mexico. *Selbyana* 27:54-59.

García-Suárez MD, Rico-Gray V, Serrano H. 2003. Distribution and abundance of *Tillandsia* spp. (Bromeliaceae) in the Zapotitlan Valley, Puebla, Mexico. *Plant Ecology* 166:207-215.

Garth RE. 1964. The ecology of spanish moss (*Tillandsia usneoides*): its growth and distribution. *Ecology* 45:470-481.

Geraldino HCL, Caxambu MG, de Souza DC. 2010. Composição florística e estrutura da comunidade de epífitas vasculares em uma área de ecótono em Campo Mourão, PR, Brasil. *Acta Botanica Brasílica* 24:469-482.

Ghazoul J, Sheil D. 2010. *Tropical rain forest - Ecology, diversity, and conservation,* New York: Oxford University Press.

Gonzalez-Astorga JG, Cruz-Angon A, Flores-Palacios A, Vovides AP. 2004. Diversity and genetic structure of the Mexican endemic epiphyte *Tillandsia achyrostachys* E. Morr. ex Baker var. achyrostachys (Bromeliaceae). *Annals of Botany* 94:545-551.

Gottsberger G, Morawetz W. 1993. Development and distribution of the epiphytic flora in an Amazonian savanna in Brazil. *Flora* 188:145-151.

Gowland KM, van der Merwe MM, Linde CC, Clements MA, Nicotra AB. 2013. The host bias of three epiphytic Aeridinae orchid species is reflected, but not explained, by mycorrhizal fungal associations. *American Journal of Botany* 100:764-777.

Gowland KM, Wood J, Clements MA, Nicotra AB. 2011. Significant phorophyte (substrate) bias is not explained by fitness benefits in three epiphytic orchid species. *American Journal of Botany* 98:197-206.

Haro-Carrión X. 2004. Bromeliad distribution in two plots in the Sumaco biosphere reserve. *Lyonia* 7:57-62.

Harshani HBC, Senanayake SP, Sandamali H. 2014. Host tree specificity and seed germination of Dendrobium aphyllum (Roxb.) CEC Fisch in Sri Lanka. *Journal of the National Science Foundation of Sri Lanka* 42:71-86.

Hassall DC, Kirkpatrick JB. 1985. The diagnostic value and host relationships of the dependent synusia in the forest of Mount Korobaba, Fiji. *New Zealand Journal of Botany* 23:47-54.

Hedge SN. 1985. Observations on the habitat distribution of orchids of Arunachal Pradesh, India. *Journal Of The Bombay Natural History Society* 82:114-129.

Hemp A. 2001. Life forms and strategies of forest ferns on Mt. Kilimanjaro. In: Gottsberger G, Liede S eds. *Life forms and dynamics in tropical forests.* Stuttgart: J. Cramer, 95-130

Henderson MR. 1935. The epiphyte flora of *Dipterocarpus oblongifolius* Bl. ("Neram"). *Gardens' Bulletin, Singapore* 9:93-97.

Hernández-Rosas J, Carlsen M. 2003. Estructura de la sinusia de plantas del dosel en un portador (*Eschweilera parviflora*, Lecythidaceae) del bosque húmedo tropical del Alto Orinoco, Edo. Amazonas, Venezuela. *Ecotrópicos* 16:33-41.

Hernández JA, Diaz MA. 2000. A new species of *Tetramicra* (Orchidaceae) from eastern Cuba. *Harvard Papers in Botany* 5:189-192.

Herndon C. 1996. Cymbidiella: a study in contrasts. *Orchids* 65:390-397.

Hietz P, Hietz-Seifert U. 1995a. Composition and ecology of vascular epiphyte communities along an altitudinal gradient in central Veracruz, Mexico. *Journal of Vegetation Science* 6:487-498.

Hietz P, Hietz-Seifert U. 1995b. Structure and ecology of epiphyte communities of a cloud forest in central Veracruz, Mexico. *Journal of Vegetation Science* 6:719-728.

Hirata A, Kamijo T, Saito S. 2009. Host trait preferences and distribution of vascular epiphytes in a warm-temperate forest. *Plant Ecology* 201:247-254.

Husk GJ, Weishampel JE, Schlesinger WH. 2004. Mineral dynamics in Spanish moss, *Tillandsia usneoides* L. (Bromeliaceae), from Central Florida, USA. *Science of the Total Environment* 321:165-172.

Ibisch PL. 1996. *Neotropische Epiphytendiversität - das Beispiel Bolivien,* Wiehl: Martina Galunder-Verlag.

Ibisch PL, Rauer G, Rudolph D. 1995. The pantropical epiphyte *Ophioglossum palmatum* (Ophioglossaceae: Pteridophyta), a new record for Bolivia. *Fern Gazette* 15:75-76.

Ipulet P, Kasenene J. 2008. Diversity of genus *Ficus* L. (Moraceae) in farmlands and pastoral areas in Buganda region, central Uganda. *African Journal of Ecology* 46:52-58.

Janzen DH. 1969. Allelopathy by myrmecophytes: the ant *Azteca* as an allelopathic agent of *Cecropia*. *Ecology* 50:147-153.

Jarman SJ, Kantvilas G, Brown MJ. 1986. The ecology of pteridophytes in Tasmanian cool temperate rainforest. *Fern Gazette* 13:77-86.

Johansson D. 1974. Ecology of vascular epiphytes in West African rain forests. *Acta Phytogeographica Suecica* 59:1-129.

Johnson A, Awan B. 1972. The distribution of epiphytes on *Fagraea fragrans* and *Swietenia macrophylla*. *Malayan Forester* 35:5-12.

Kaufmann E, Weissflog A, Hashim R, Maschwitz U. 2001. Ant-Gardens on the giant bamboo *Gigantochloa scortechinii* (Poaceae) in West-Malaysia. *Insectes Sociaux* 48:125-133.

Kennedy GC. 1972. Notes on the genera *Cymbidiella* and *Eulophiella* of Madagascar. *Orchid Digest* 36:121-122.

Kersten RD, Borgo M, Silva SM. 2009. Diversity and distribution of vascular epiphytes in an insular Brazilian coastal forest. *Revista de Biología Tropical* 57:749-759.

Kiew R, Anthonysamy S. 1987. A comparative study of vascular epiphytes in three epiphyte-rich habitats at Ulu Endau, Johore, Malaysia. *Malayan Nature Journal* 41:303-315.

Kolbek J. 1995. Notes on epiphytic communities in forests of North Korea. *Preslia, Praha* 67:41-45.

Köster N, Nieder J, Barthlott W. 2011. Effect of host tree traits on epiphyte diversity in natural and anthropogenic habitats in Ecuador. *Biotropica* 43:685-694.

Kundu SR, Pal M. 1998. An autecological investigation of *Pyrrosia adnascens* (sw.) Ching (Polypodiaceae) in Indian Botanic Garden, Howrah. *Journal of Economic and Taxonomic Botany* 22:241-245.

La Croix IF, La Croix EAS, La Croix TM. 1991. *Orchids of Malawi: the epiphytic and terrestrial orchids from South and East Central Africa,* Rotterdam: Taylor & Francis.

Laube S, Zotz G. 2006. Neither host-specific nor random: vascular epiphytes on three tree species in a Panamanian lowland forest. *Annals of Botany* 97:1103-1114.

Lecoufle M. 1964. Notes about *Cymbidiella*. *Orchid Review* 72:233-236.

Li R, Dao Z-L. 2014. A new species of Coelogyne (Orchidaceae) from western Yunnan, China. *Phytotaxa* 162:115-119.

López-Villalobos A, Flores-Palacios A, Ortiz-Pulido R. 2008. The relationship between bark peeling rate and the distribution and mortality of two epiphyte species. *Plant Ecology* 198:265-274.

Malizia A. 2003. Host tree preference of vascular epiphytes and climbers in a subtropical montane cloud forest of Northwest Argentina. *Selbyana* 24:196-205.

Martin CE, Lin T-C, Hsu C-C, Lin S-H. 2007. No effect of host tree species on the physiology of the epiphytic orchid *Bulbophyllum japonicum* in a subtropical rainforest in Northeastern Taiwan. *Taiwan Journal of Forest Science* 22:241-251.

Martínez-Meléndez N, Pérez-Farrera MA, Flores-Palacios A. 2008. Vertical stratification and host preference by vascular epiphytes in a Chiapas, Mexico, cloud forest. *Revista De Biologia Tropical* 56:2069-2086.

Matias LQ, Braga PIS, Freire AG. 1996. Reproductive biology of *Constantia cipoensis* Porto and Brade (Orchidaceae), an endemic species from the Serra do Cipo, Minas Gerais. *Revista Brasileira de Botânica* 19:119-125.

Mayo SJ, Félix LP, Jardim JG, Carvalho AM. 2000. *Anthurium bromelicola* - a remarkable new species from Northeast Brazil. *Aroideana* 23:89-99.

Medeiros AC, Loope LL, Anderson SJ. 1993. Differential colonization of epiphytes on native (*Cibotium* spp.) and alien (*Cyathea cooperi*) tree ferns in a Hawaiian rain forest. *Selbyana* 14:71-74.

Medeiros TDS, Gonçalves Jardim MA. 2011. Distribuição vertical de orquídeas epífitas na Área de Proteção Ambiental (APA) Ilha do Combu, Belém, Pará, Brasil. *Revista Brasileira de Biociências* 9:33-38.

Mehltreter K. 2008. Phenology and habitat specificity of tropical ferns. In: Ranker TA, Haufler CH eds. *Biology and evolution of ferns and lycophytes.* New York: Cambridge University Press, 201-221

Mehltreter K, Flores-Palacios A, García-Franco JG. 2005. Host preferences of low-trunk vascular epiphytes in a cloud forest of Veracruz, Mexico. *Journal of Tropical Ecology* 21:651-660.

Merwin MC, Rentmeester SA, Nadkarni NM. 2003. The influence of host tree species on the distribution of epiphytic bromeliads in experimental monospecific plantations, La Selva, Costa Rica. *Biotropica* 35:37-47.

Mesler MR. 1975. The gametophytes of *Ophioglossum palmatum* L. *American Journal of Botany* 62:982-992.

Mickel JT, Beitel JM. 1988. Pteridophyte flora of Oaxaca, Mexico. *Memoirs of the New York Botanical Garden* 46:412-567.

Migenis LE, Ackerman JD. 1993. Orchid-phorophyte relationships in a forest watershed in Puerto Rico. *Journal of Tropical Ecology* 9:231-240.

Mondragón D, Durán R, Ramírez I, Olmsted IC. 1999. Population dynamics of *Tillandsia brachycaulos* Schltdl. (Bromeliaceae) in Dzibilchaltun National park, Yucatán. *Selbyana* 20:250-255.

Mondragón D, Durán R, Ramírez I, Valverde T. 2004. Temporal variation in the demography of the clonal epiphyte *Tillandsia brachycaulos* (Bromeliaceae) in the Yucatán Peninsula, Mexico. *Journal of Tropical Ecology* 20:189-200.

Moran R, Riba R. 1995. Psilotaceae a Salviniaceae. In: Davidse G, Sousa MS, Chater AO eds. *Flora Mesoamericana.* Universidad Nacional Autónoma de México, Instituto de Biología.

Moran RC, Klimas S, Carlsen M. 2003. Low-trunk epiphytic ferns on tree ferns versus angiosperms in Costa Rica. *Biotropica* 35:48-56.

Moran RC, Russell RV. 2004. The occurrence of *Trichomanes godmanii* (Hymenophyllaceae) on *Welfia georgii* (Arecaceae) at the La Selva Biological Station, Costa Rica. *American Fern Journal* 94:70-76.

Morris B. 1968. The epiphytic orchids of the Shire Highlands, Malawi. *Proceedings of the Linnean Society* 179:51-66.

Morris B. 1970. *The epiphytic orchids of Malawi,* Bulawayo: The Society of Malawi.

Mucunguzi P. 2008. Diversity and distribution of epiphytic orchids in Kibale National Park, Uganda. *Selbyana* 29:217-225.

Muñoz AA, Chacon P, Perez F, Barnert ES, Armesto JJ. 2003. Diversity and host tree preferences of vascular epiphytes and vines in a temperate rainforest in southern Chile. *Australian Journal of Botany* 51:381-391.

Mursidawati S, Norton DA, Astuti IP. 1999. Distribution of *Pomatocalpa spicata* Breda (Orchidaceae) within and among host trees in Manusela National Park, Seram, Maluku Archipelago, Indonesia. *Selbyana* 20:116-119.

Neger FW. 1913. *Biologie der Pflanzen auf experimenteller Grundlage,* Stuttgart: Verlag von Ferdinand Enke.

Nieder J, Engwald S, Klawun M, Barthlott W. 2000. Spatial distribution of vascular epiphytes (including hemiepiphytes) in a lowland Amazonian rain forest (Surumoni crane plot) of southern Venezuela. *Biotropica* 32:385-396.

Nieder J, Prosperi J, Michaloud G. 2001. Epiphytes and their contribution to canopy diversity. *Plant Ecology* 153:51-63.

Nir MA. 1988. The survivors: orchids on a Puerto Rican coffee finca. *American Orchid Society Bulletin* 57:989-995.

Noumi VN, Zapfack L, Sonke B, Achoundong G, Kengne OC. 2010. Distribution et richesse taxonomiques des épiphytes de quelques phorophytes au Parc national de Korup (Cameroun). *International Journal of Environmental Studies* 67:51-61.

Obermüller FA, Silveira M, Salimon CI, Daly DC. 2012. Epiphytic (including hemiepiphytes) diversity in three timber species in the southwestern Amazon, Brazil. *Biodiversity and Conservation* 21:565-575.

Oliver WRB. 1930. New Zealand epiphytes. *Journal of Ecology* 18:1-50.

Olson DM, Dinerstein E, Wikramanayake ED, Burgess ND, Powell GVN, Underwood EC, D'Amico JA, Itoua I, Strand HE, Morrison JC, Loucks CJ, Allnutt TF, Ricketts TH, Kura Y, Lamoreux JF, Wettengel WW, Hedao P, Kassem KR. 2001. Terrestrial ecoregions of the worlds: A new map of life on Earth. *BioScience* 51:933-938.

Otero JT, Aragón S, Ackerman JD. 2007. Site variation in spatial aggregation and phorophyte preference in *Psychilis monensis* (Orchidaceae). *Biotropica* 39:227-231.

Pessin LJ. 1925. An ecological study of the polypody fern *Polypodium polypodioides* as an epiphyte in Mississippi. *Ecology* 6:17-38.

Piazzon M, Larrinaga AR, Santamaria L. 2011. Are nested networks more robust to disturbance? A test using epiphyte-tree, comensalistic networks. <Go to ISI>://WOS:000290483600018.

Piers F. 1968. *Orchids of East Africa,* Lehre: Cramer.

Pollard G. 1973. La opinión de un hombre. *Orquidea* 3:184-190.

Porembski S. 1996. Notes on the vegetation of inselbergs in Malawi. *Flora* 191:1-8.

Porembski S. 2003. Epiphytic orchids on arborescent Velloziaceae and Cyperaceae: Extremes of phorophyte specialisation. *Nordic Journal of Botany* 23:505-512.

Pridgeon AM. 2005. *Epidendroideae (Part one),* Oxford: Oxford University Press.

Proctor GR. 1989. *Ferns of Puerto Rico and the Virgin Islands*: New York Botanical Garden Press Dept.

Raventós J, Mujica E, Wiegand T, Bonet A. 2011. Analyzing the spatial structure of *Broughtonia cubensis* (Orchidaceae) populations in the dry forests of Guanahacabibes, Cuba. *Biotropica* 43:173-182.

Reiche K. 1907. *Grundzüge der Pflanzenverbreitung in Chile,* Leipzig: Verlag von Wilhelm Engelmann.

Reif A, Allen RB. 1988. Plant communities of the steepland conifer-broadleaved hardwood forest of central Westland, South Island, New Zealand. *Phytocoenologia* 16:145-224.

Reyes-García C, Griffiths H, Rincón E, Huante P. 2008. Niche differentiation in tank and atmospheric epiphytic bromeliads of a seasonally dry forest. *Biotropica* 40:168-175.

Riveros M, Ramírez C. 1978. Fitocenoses epífitas de la asociación *Lapagerio-Aextoxiconetum* en el fundo San Martín (Valdivia-Chile). *Acta Científica Venezolana* 29:163-169.

Roberts NR, Dalton PJ, Jordan GJ. 2005. Epiphytic ferns and bryophytes of Tasmanian tree-ferns: A comparison of diversity and composition between two host species. *Austral Ecology* 30:146-154.

Rupp HMR, McGillivray DJ. 1969. *The orchids of New South Wales,* Sydney: National Herbarium of N.S.W.

Sanford WW. 1968. Distribution of epiphytic orchids in semi-deciduous tropical forest in southern Nigeria. *Journal of Ecology* 56:697-705.

Sanford WW. 1974. The ecology of orchids. In: Withner CL ed. *The orchids. Scientific Studies.* New York: J. Wiley and Sons, 1-100

Sáyago R, Lopezaraiza-Mikel M, Quesada M, Álvarez-Anorve MY, Cascante-Marín A, Bastida JM. 2013. Evaluating factors that predict the structure of a commensalistic epiphyte-phorophyte network. *Proceedings of the Royal Society B: Biological Sciences* 280:20122821.

Schimper AFW. 1888. *Die epiphytische Vegetation Amerikas,* Jena: Gustav Fischer.

Schlesinger WJ, Marks PL. 1977. Mineral cycling and the niche of Spanish moss, *Tillandsia usneoides* L. *American Journal of Botany* 64:1254-1262.

Schmitt J, Windisch P. 2010. Biodiversity and spatial distribution of epiphytic ferns on *Alsophila setosa* Kaulf. (Cyatheaceae) caudices in Rio Grande do Sul, Brazil. *Brazilian Journal of Biology* 70:521-528.

Schmitt JL, Windisch PG. 2005. Aspectos ecológicos de *Alsophila setosa* Kaulf. (Cyatheaceae, Pteridophyta) no Rio Grande do Sul, Brasil. *Acta Botanica Brasílica* 19:859-865.

Schnell R. 1970. *Introduction à la phytogéographie de pays tropicaux. 1. Les problèmes généraux: la flore et les structures,* Paris: Gauthier-Villars.

Scott GAM, Rowley JA. 1975. A lowland vegetation sequence in South Westland. Part 2. Ground and epiphytic vegetation. *Proceedings of the New Zealand Ecological Society* 22:93-108.

Sehgal RN, Mehra PN. 1984. Distribution pattern of orchids in Khasi and Jaintia India hills. *Indian Journal of Forestry* 7:114-119.

Sehnem A. 1977. As filicíneas do Sul do Brasil, sua distribuição geográfica, sua ecologia e suas rotas de imigração. *Pesquisas Botânica* 31:1-108.

Sharma A. 2010. *Diversity, distribution pattern and host specificity of epiphytic orchids along Bhote Koshi gorge (Upper Tamakoshi Valley), Dolakha, Central Nepal,* MSc MSc Thesis, Tribhuvan University, Kirtipur Kathmandu.

Silva IA, Ferreira AWC, Lima MIS, Soares JJ. 2010. Networks of epiphytic orchids and host trees in Brazilian gallery forests. *Journal of Tropical Ecology* 26:127-137.

Song XQ, Meng QW, Wing YT, Luo YB. 2009. *Thrixspermum odoratum* (Orchidaceae), a new species from Hainan Island, China. *Annales Botanici Fennici* 46:595-598.

Sonké B, Zapfack L, Folefack DC. 2001. Distribution des epiphytes vasculaires sur les haies vives dans la region de Bafou (Cameroun). *Systematics and Geography of Plants* 71:209-222.

Sosa V, Platas T. 1998. Extinction and persistence of rare orchids in Veracruz, Mexico. *Conservation Biology* 12:451-455.

Soto Arenas MA. 1994. Population studies in Mexican orchids. In: Pridgeon A ed. *Proceedings of the 14th world orchid conference.* London: HMSO, 153-160

Sulit MD. 1950. Field observations on tree hosts of orchids in the Philippines. *Philippine Orchid Review* 3:3-8.

Sulit MD. 1953. Field observations on tree hosts of orchids in Palawan. *Phillipine Orchid Review* 5:16.

ter Steege H, Cornelissen JHC. 1989. Distribution and ecology of vascular epiphytes in lowland rain forest of Guyana. *Biotropica* 21:331-339.

Tomazini V. 2007. *Estrutura de epífitas vasculares e de forófitos em formação florestal ripária do Parque Estadual do Rio Ivinhema, Estado de Mato Grosso do Sul, Brasil,* PhD Thesis, Universidade Estadual de Maringá, Brazil.

Trapnell DW, Hamrick JL. 2006. Variety of phorophyte species colonized by the neotropical epiphyte, *Laelia rubescens* (Orchidaceae). *Selbyana* 27:60-64.

Traxmandlová I, Bhattarai B, Kindlmann P. 2012. Orchid diversity in the Chitwan district. In: Kindlmann P ed. *Himalayan biodiversity in the changing world.* Springer Netherlands, 71-95

Tremblay RL, Zimmerman JK, Lebrón L, Bayman P, Sastre I, Axelrod F, Alers-García J. 1998. Host specificity and low reproductive success in the rare endemic Puerto Rican orchid *Lepanthes caritensis*. *Biological Conservation* 85:297-304.

Valdivia PE. 1977. Estudio botánico y ecológico de la región del Río Uxpanapa, Veracruz. N^o^ 4. Las epífitas. *Biotica* 2:55-81.

Valencia-Díaz S, Corona-López A, Toledo-Hernández V, Flores-Palacios A. 2014. Is branch damage by xylophages related to the presence of epiphytes? *Arthropod-Plant Interactions* 8:25-32.

Valencia-Díaz S, Flores-Palacios A, Rodríguez-López V, Ventura-Zapata E, Jiménez-Aparicio AR. 2010. Effect of host-bark extracts on seed germination in *Tillandsia recurvata*, an epiphytic bromeliad. *Journal of Tropical Ecology* 26:571-581.

Válka Alves RJ, Kolbek J, Becker J. 2008. Vascular epiphyte vegetation in rocky savannas of southeastern Brazil. *Nordic Journal of Botany* 26:101-117.

Van den Berg C, Smidt EC, Marcal S. 2006. *Leptotes vellozicola*: A new species of Orchidaceae from Bahia, Brazil. *Neodiversity* 1:1-5.

Van Oye MP. 1924. Sur l'écologie des épiphytes a la surface des troncs d'arbres à Java. *Revue Génerale de Botanique* 36:12-30 and 68-83.

Vergara-Torres CA, Pacheco-Alvarez MC, Flores-Palacios A. 2010. Host preference and host limitation of vascular epiphytes in a tropical dry forest of central Mexico. *Journal of Tropical Ecology* 26:563-570.

Waechter JL, Baptista LRM. 2004. Abundância e distribuição de orquídeas epifítas em uma floresta turfosa do Brasil Meridional. In: Barros F, Kerbauy GB eds. *Orquideologia sul-americana: uma compilacao cientifica.* Sao Paulo, 135-145

Wallace BJ. 1981. *The Australian vascular epiphytes: flora and ecology,* PhD PhD Thesis, University of New England, Australia.

Watthana S. 2004. Ecology and conservation biology of *Pomatocalpa naevata* J.J. Sm. (Orchidaceae). *Natural History Bulletin of the Siam Society* 52:201-215.

Watthana S, Pedersen HÆ. 2008. Phorophyte diversity, substrate requirements and fruit set in *Dendrobium scabrilingue* Lindl. (Asparagales: Orchidaceae): Basic observations for re-introduction experiments. *The Natural History Journal of Chulalongkorn University* 8:135-142.

Wee YC. 1978. Vascular epiphytes of Singapore's wayside trees. *Gardens' Bulletin, Singapore* 31:114-126.

Went FW. 1940. Soziologie der Epiphyten eines tropischen Regenwaldes. *Annales du Jardin Botanique de Buitenzorg* 50:1-98.

Wolf JHD. 1994. Factors controlling the distribution of vascular and non-vascular epiphytes in the northern Andes. *Vegetatio* 112:15-28.

Wyse SV, Burns BR. 2011. Do host bark traits influence trunk epiphyte communities? *New Zealand Journal of Botany* 35:296-301.

Xu HQ, Liu WY. 2005. Species diversity and distribution of epiphytes in the montane moist evergreen broad-leaved forest in Ailao Mountain, Yunnan. *Biodiversity Science* 13:137-147.

Zapfack L, Nkongmeneck AB, Villiers JF, Lowman MD. 1996. The importance of pteridophytes in the epiphytic flora of some phorophytes of the Cameroonian semi-deciduous rain forest. *Selbyana* 17:76-81.

Zhang LW, Nurvianto S, Harrison R. 2010. Factors affecting the distribution and abundance of *Asplenium nidus* L. in a tropical lowland rain forest in Peninsular Malaysia. *Biotropica* 42:464-469.

Zimmerman JK, Olmsted IC. 1992. Host tree utilization by vascular epiphytes in a seasonally inundated forest (Tintal) in Mexico. *Biotropica* 24:402-407.

Zotz G. 2002. Gefässepiphyten in temperaten Wäldern. *Bauhinia* 16:13-22.

Zotz G. 2005. Vascular epiphytes in the temperate zones - a review. *Plant Ecology* 176:173-183.

Zotz G, Bermejo P, Dietz H. 1999. The epiphyte vegetation of *Annona glabra* on Barro Colorado Island, Panama. *Journal of Biogeography* 26:761-776.

Zotz G, Mendieta-Leiva G, Wagner K. 2014. Vascular epiphytes at the treeline - composition of species assemblages and population biology. *Flora* 209:385-390.

Zotz G, Schultz S. 2008. The vascular epiphytes of a lowland forest in Panama-species composition and spatial structure. *Plant Ecology* 195:131-141.

Zotz G, Vollrath B. 2003. The epiphyte vegetation of the palm *Socratea exorrhiza* - correlations with tree size, tree age and bryophyte cover. *Journal of Tropical Ecology* 19:81-90.
